# The spatial and temporal organization of origin firing during the S-phase of fission yeast

**DOI:** 10.1101/gr.180372.114

**Published:** 2015-03

**Authors:** Atanas Kaykov, Paul Nurse

**Affiliations:** 1 The Rockefeller University, New York, New York 10065, USA;; 2 The Francis Crick Institute, Lincoln’s Inn Fields Laboratories, London WC2A 3LY, United Kingdom

## Abstract

Eukaryotes duplicate their genomes using multiple replication origins, but the organization of origin firing along chromosomes and during S-phase is not well understood. Using fission yeast, we report the first genome-wide analysis of the spatial and temporal organization of replication origin firing, analyzed using single DNA molecules that can approach the full length of chromosomes. At S-phase onset, origins fire randomly and sparsely throughout the chromosomes. Later in S-phase, clusters of fired origins appear embedded in the sparser regions, which form the basis of nuclear replication foci. The formation of clusters requires proper histone methylation and acetylation, and their locations are not inherited between cell cycles. The rate of origin firing increases gradually, peaking just before mid S-phase. Toward the end of S-phase, nearly all the available origins within the unreplicated regions are fired, contributing to the timely completion of genome replication. We propose that the majority of origins do not fire as a part of a deterministic program. Instead, origin firing, both individually and as clusters, should be viewed as being mostly stochastic.

Eukaryotic DNA replication occurs during S-phase of the cell cycle when DNA synthesis initiates at multiple replication origins located throughout the chromosomes. In most eukaryotic organisms, origins are not defined by a specific DNA consensus sequence, although they are usually AT rich and are used inefficiently (Mechali 2010). Investigations of origin firing on single DNA molecules have led some to suggest that origins fire randomly along chromosomes, whereas others propose that origins fire in clusters (Jackson and Pombo 1998; Blow et al. 2001; Pasero et al. 2002; Patel et al. 2006; Czajkowsky et al. 2008; Demczuk et al. 2012). Studies at the level of the whole nucleus showed that DNA synthesis occurs in replication foci enriched in replication factors (Berezney et al. 2000). Histone modifications have been shown to play a role in the timing and efficiency of origin firing (Vogelauer et al. 2002; Aggarwal and Calvi 2004; Tabancay and Forsburg 2006; Knott et al. 2009). In general, euchromatic regions are more enriched with origins that fire early in S-phase, and heterochromatic regions are enriched with origins that fire late. Despite extensive studies of origin firing and its molecular basis (Bell and Dutta 2002; Sherstyuk et al. 2013), a number of important questions remain unanswered. A global description of origin firing along chromosomes as cells proceed through S-phase is lacking for any eukaryote; the extent to which origins are randomly distributed or are clustered is not known; it is not clear how many origins are organized into a strictly deterministic program of origin firing; it remains uncertain how the pattern of origin firing contributes to the completion of DNA replication; and the relationship of origins with nuclear replication foci is unclear. Here we address these questions in the fission yeast *Schizosaccharomyces pombe* by analyzing long single DNA molecules that can approach the full length of chromosomes.

The fission yeast is suitable for these investigations because it has a small 13.6-Mb genome distributed between only three chromosomes that are 5.6 Mb, 4.5 Mb, and 3.5 Mb in length (Wood et al. 2002). Replication origins are AT-rich but are not defined by a strict consensus sequence, and at least half of the intergenic regions have potential origin activity (Dai et al. 2005). A total of 401 strong origins with firing efficiencies >10% have been mapped using microarray-based analyses (Heichinger et al. 2006; Hayashi et al. 2007). These origins have a mean inter-origin distance of 31 kb and efficiencies ranging from 10% to 80%. A further 503 weak origins (with efficiencies <10%) have been mapped, giving a total of around 900 origins distributed along chromosomes with an average inter-origin distance of 14 kb (Heichinger et al. 2006). Pulse labeling and DNA combing of short (200–500 kb) single DNA molecules have revealed that origin firing is randomly distributed along chromosomes without the formation of clusters (Patel et al. 2006). This contrasts with studies of short DNA molecules in Metazoa which indicate that origins are activated in clusters (Jackson and Pombo 1998; Blow et al. 2001). Studies of the temporal organization of origin firing in fission yeast have shown that origins near the centromeres and mating type region fire early in S-phase, whereas origins in subtelomeric regions fire late in S-phase (Kim and Huberman 2001; Hayashi et al. 2009; Hayano et al. 2012; Tazumi et al. 2012). Replication foci have been identified using PCNA-GFP, and up to 16 foci have been imaged in a fission yeast nucleus (Meister et al. 2007). However, despite this extensive work, we lack a comprehensive understanding of the spatial and temporal organization of replication origin firing on individual chromosomes in fission yeast or any eukaryote.

By analysis of long single DNA molecules representing significant segments of individual chromosomes, we have made a genome-wide description of origin firing during S-phase. This has allowed us to address how origin firing is distributed along chromosomes, the program of origin firing during S-phase, the organization of origin clusters, and the relationship of clusters of fired origins to nuclear replication foci.

## Results

### Origin firing on long single DNA molecules

**Figure 1.**
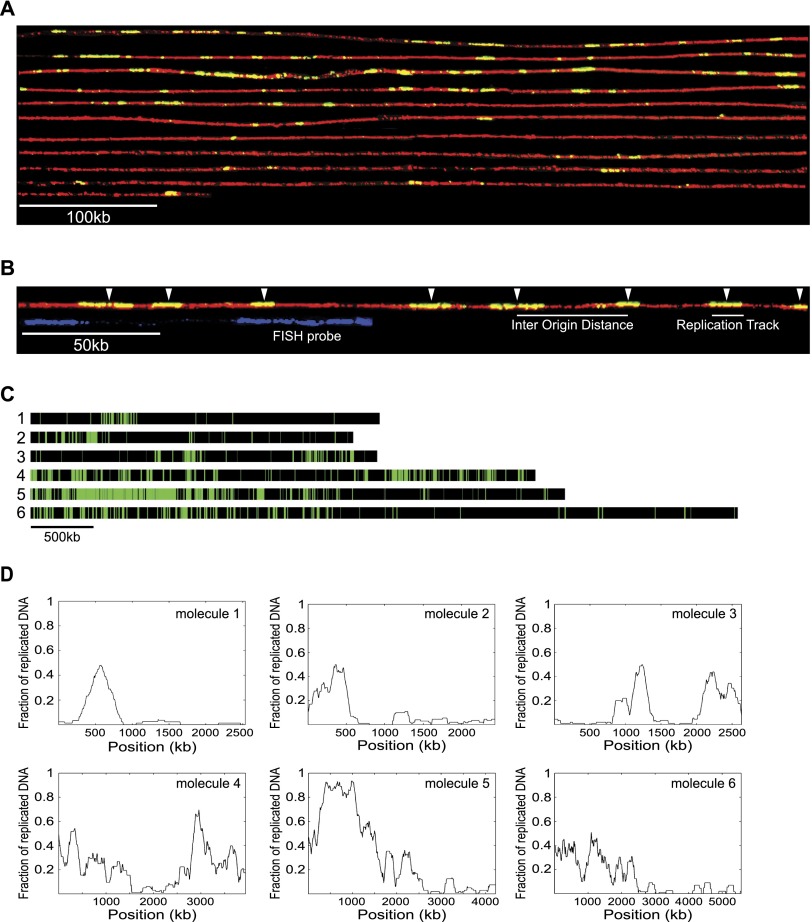
Visualizing DNA replication on single DNA molecules using DNA combing. (*A*) A single 5.6-Mb molecule that approximately spans the entire length of Chromosome I. To visualize individual BrdU tracks, the molecule was “cut” in silico into 21 consecutive fragments of 270 kb each, and a composite image was constructed. Newly synthesized DNA was labeled in vivo using the halogenated thymidine analog BrdU. Protein-free DNA was combed onto a silanized glass surface and visualized with anti-thymidine antibody (red), and incorporated BrdU was visualized with anti-BrdU antibody (green). BrdU tracks (replication tracks) shown in yellow (green overlaying red) correspond to newly synthesized DNA. (*B*) White arrows point to the *middle* of replication tracks (shown with a short white line *below* the DNA molecule) where we assume replication origins are located. The distance between the middle of two adjacent replication tracks represents the inter-origin distance (IOD) (shown with a longer white line below the DNA molecule). To detect and orient specific DNA sequences on combed DNA molecules, we used fluorescent in situ hybridization (FISH). Each chromosomal position is detected by a unique signature of two probes with differing lengths and distances between them (shown in blue *below* the DNA molecule), which allowed us to orient the combed DNA molecules and to align them to the corresponding chromosomal region. (*C*) The distribution of replication tracks on six single DNA molecules randomly selected from the population of molecules. Green bars represent BrdU replication tracks and black bars represent unreplicated segments on DNA molecules. Black and green bars are drawn to scale. All DNA molecules analyzed in this study are represented in Supplemental Figure S15, and the measurements for each molecule are listed in Supplemental Table S2. The DNA molecule shown in *A* corresponds to molecule 6 in *C*. (*D*) Moving average analysis using a 200-kb window size in 2-kb steps (the resolution of the DNA combing technique) of the six molecules shown in *C*. Each value corresponds to the fraction of replicated DNA within a 200-kb stretch of DNA. Peaks represent chromosomal domains rich in replicated DNA, whereas troughs represent chromosomal domains poor in replicated DNA.

We synchronized fission yeast cells for entry into S-phase and pulse-labeled newly synthesized DNA with nucleotide analogs such as BrdU and EdU (Supplemental Fig. S1). Genomic DNA was then combed onto glass surfaces and immunodetected. Previously, DNA combing has been restricted to molecules of 200–500 kb in length (Michalet et al. 1997; Pasero et al. 2002; Patel et al. 2006; Czajkowsky et al. 2008; Tuduri et al. 2010). By gently preparing DNA molecules for combing, we could routinely analyze molecules of 1.5–2.5 Mb and occasionally up to 5 Mb. Figure 1A shows an example of a single DNA molecule that approximately spans the entire length of the 5.6-Mb Chromosome I. Newly synthesized DNA incorporating BrdU is shown in green, with whole molecules counterstained with anti-thymidine antibody shown in red. Replication origins were mapped in the middle of individual replication tracks corresponding to replicons, and inter-origin distances (IODs) were estimated by the distances between midpoints of two adjacent replication tracks (Fig. 1B). To minimize the risk of two or more origins being responsible for a single replication track segment, pulse lengths were kept short, only molecules <50% replicated were analyzed, and IODs were measured using replication tracks <20 kb (by far the majority). We used FISH to identify chromosomal regions being analyzed (Fig. 1B). To determine the variation in the extension of combed DNA molecules, we measured the length of the same FISH probe in 39 different molecules (Supplemental Fig. S14). The average size of the measured probe was 146 kb (SD = 15 kb) compared to an expected size of 150 kb, demonstrating that the DNA combing technique allows consistent measurements of adjacent inter-origin distances.

### Origin firing distribution is clustered

**Figure 2.**
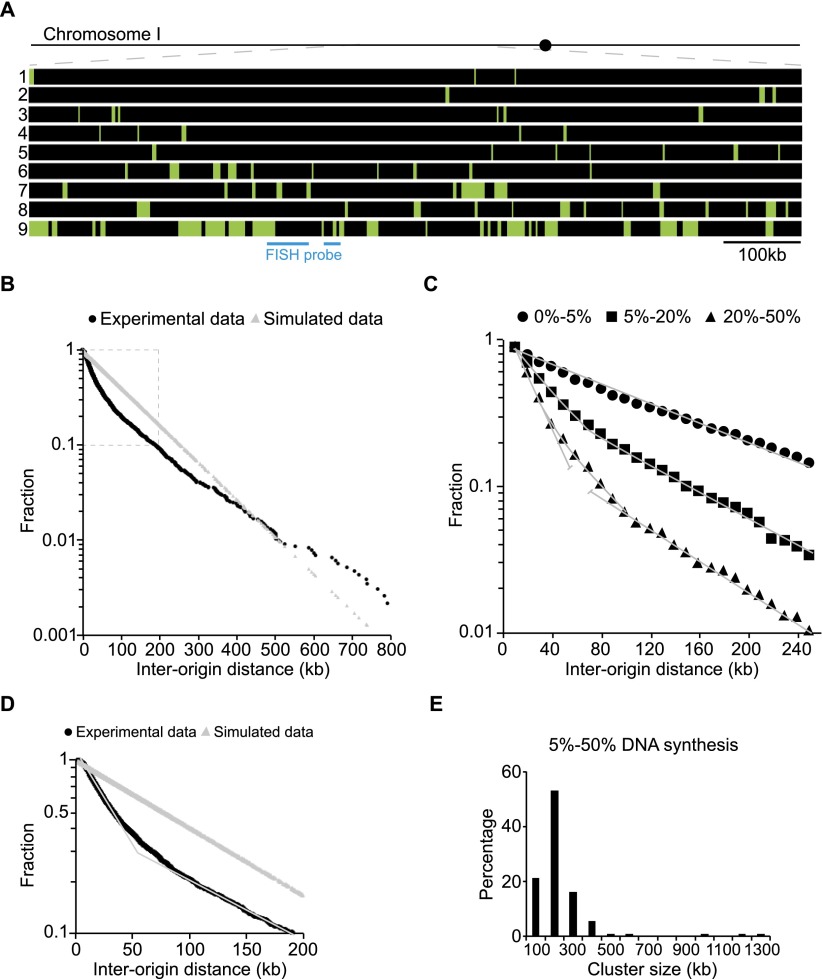
Replication origins both fire stochastically and form clusters. (*A*) Replication profile of nine completely overlapping 1-Mb single DNA molecules corresponding to the same region on Chromosome I (from 2100 to 3100 kb). (*B*) Semilog plot of the cumulative frequency of IODs measured for 131 single DNA molecules with an average length of 1.8 Mb and replicated up to 50% (black curve). The values on the *y*-axis correspond to the fraction of IODs that are larger in size than the corresponding IOD on the *x*-axis. The gray line corresponds to simulated data for a purely random distribution of the same number of origins over the same DNA length. The experimental measurements deviate significantly from the purely stochastic prediction, *P* < 0.001 (Lilliefors statistical test). (*C*) Semilog plot of the cumulative frequencies of IODs for molecules sampled according to their extent of DNA synthesis; black circles, black squares, and black triangles correspond to molecules replicated from 0%–5%, 5%–20%, and 20%–50%, respectively. At the beginning of S-phase, origin selection is random, as the cumulative frequency of IODs for molecules replicated up to 5% fits a straight line. For molecules replicated from 5%–20%, the curve deviates from the straight line at short IODs. The curve corresponding to molecules replicated from 20% to 50% can be decomposed into two straight lines with different slopes. (*D*) Semilog plot of the data within the dashed box in *B*, representing 90% of the IODs. The experimental data curve can be decomposed into two straight lines with different slopes (thin gray line), indicative of two regimes of stochastic origin selection operating along chromosomes. (*E*) Histogram of cluster sizes for molecules replicated from 5% to 50%.

The distribution of origin firing throughout the genome was determined by analyzing 131 single molecules with an average length of 1.8 Mb. To assess if origins were randomly fired along the molecules, a cumulative frequency semilog plot was constructed of IODs. If origin distribution was random, a straight line would describe the cumulative frequency of IODs. Figure 2B and D shows the experimentally measured IODs deviated from a straight line. Instead, the curve can be represented by two domains that can be fitted with two straight lines with steeper and shallower slopes (Fig. 2D). The first domain represented by the initial steep region covers 50%–55% of the origins and is exponential, indicative of random firing; 50% of the IODs in the domain were <17 kb. The second domain represented by the later, more shallow region, is also exponential with 50% of the IODs <160 kb. We conclude that origins are organized in clusters made up of closely spaced origins represented by the first domain, and that these are separated by regions with more sparsely spaced origins represented by the second domain.

We postulated that this pattern of origin firing might change as the chromosomes replicate. To investigate this, we divided the molecules into groups depending on the extent of their replication, 0%–5%, 5%–20%, and 20%–50%, and plotted their IOD cumulative frequencies. Molecules that were only 0%–5% replicated had origins that were randomly distributed in a similar manner along the chromosomes (Fig. 2C). Molecules more highly replicated at 5%–20% showed a slight inflexion, whereas molecules replicated 20%–50% showed two clear slopes. We tested if this inflexion of the curve can be obtained by simulating a purely stochastic model of origin firing and plotted the simulated cumulative frequencies of IODs for molecules replicated from 0%–5%, 5%–20%, and 20%–50% (Supplemental Fig. S13A,B). The data points can be easily fitted by straight lines and are significantly different from the cumulative frequencies derived from experimentally measured IODs for molecules replicated from 20%–50%. These results indicate that chromosome segments begin to replicate by firing origins that are stochastically distributed at a low frequency along chromosomes; but as replication continues, clusters of firing origins appear embedded within the background of sparser firing regions (Fig. 2C). To characterize the clusters, we examined origin distribution to estimate the maximum IOD within a cluster and the minimum number of origins defining a cluster. Given that 50% of the IODs in the clusters were <17 kb, we set the maximum IOD within a cluster at 40 kb, which provided a reasonable distinction between clusters and the sparser regions separating the clusters, where 50% of the IODs were >160 kb. We estimated the minimum number of origins defining a cluster by varying the minimum number of origins per cluster from two to 10 and setting the maximal IOD within a cluster to 40 kb. We plotted the minimum origins per cluster versus the number of clusters per genome for molecules replicated from 5%–20% or 20%–50% (Supplemental Fig. S12A,B). The plots show that both curves exhibit a steeply descending linear segment with a transition point at 4–5 or at 5–6 origins per cluster for molecules replicated from 5%–20% and 20%–50%, respectively. Given this result, we used a minimum of five origins to define a cluster; a number similar to that was reported for clusters in budding yeast, *Xenopus*, and human cells (Jackson and Pombo 1998; Blow et al. 2001; Pasero et al. 2002). To test if a minimum of five origins and a maximum IOD of 40 kb were reasonable estimates, we carried out two analyses. In the first, we analyzed the molecule shown in Figure 1A by carrying out a moving average segment window analysis measuring the amount of DNA replicated within the segments. Window sizes between 100 and 500 kb in length were used (Supplemental Fig. S2), and it can be seen that an orderly structure emerged with a window scan of 200 kb, consistent with clusters being defined minimally as having five origins no more than 40 kb apart. Similar orderly structures were revealed when similar criteria were applied to another six molecules (Fig. 1C,D). In a second analysis, we determined how the distribution of cluster size varied according to the maximum IOD applied. A maximum IOD of 40 kb produced a distribution that best represented a normal distribution (Fig. 2E), which was less obvious using IODs of 20 kb, 30 kb, 50 kb, or 60 kb (data not shown).

Using these parameters of five origins and a maximum IOD of 40 kb to analyze 63 molecules replicated from 5%–50%, we found that the average IOD within clusters was 21 kb, and that cluster size varied from 100 to 400 kb, with the most representative size being 200 kb (Fig. 2E). About 60% of all origins were located within the clusters with the remainder located in the regions between clusters. In both the clusters and the regions between clusters, firing origins were distributed stochastically, but the probability of firing was much higher in the clusters compared with the regions between clusters. We followed the temporal development of clusters as DNA replication proceeds by double labeling (Supplemental Fig. S3A). A synchronous culture was pulse-labeled with BrdU at the beginning of S-phase (Supplemental Fig. S3B, green bars), and clusters were identified as groups of five or more origins located within 40 kb of one another (Supplemental Fig. S3B, blue line). The pulse was followed by a second EdU chase for 3 min, identifying newly fired origins (Supplemental Fig. S3B, red bars). Clusters extend by firing additional origins in adjacent regions as DNA replication proceeded (Supplemental Fig. S3B, blue arrow). This result suggests local feedback, whereby clusters of origins that have fired already facilitate the firing of new origins in their vicinity.

### Patterns of origin firing and cluster formation

Next we addressed whether the firing of origins followed a program as DNA replication extends through the chromosomes. We analyzed four different regions of the genome identified using FISH probes (Supplemental Fig. S4A–D). The activation of most origins showed no evidence of a deterministic program. Origins that fired in molecules that have only just started replicating have not necessarily fired in other molecules that are more extensively replicated. These data suggest that the firing of most origins does not follow a deterministic program.

**Figure 3.**
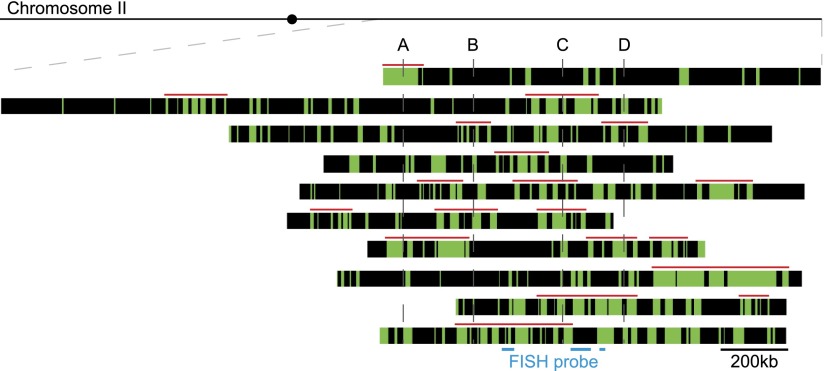
The pattern of cluster inheritance. Ten DNA molecules mapped to the corresponding sequence on Chromosome II (located from 2070 to 4420 kb). The positions of clusters of fired origins are shown as red bars on *top* of each DNA molecule. The midposition of clusters mapped on single molecules replicated <20% are named A, B, C, and D. Gray lines show positions A, B, C, and D in each molecule.

To address further the pattern of origin firing and whether clusters always appear in the same regions of the chromosomes, we combined labeling experiments with FISH to align DNA molecules according to their location and the extent of their replication. Ten molecules spanning 2.4 Mb on the right arm of Chromosome II are shown in Figure 3. On different molecules representing the same genomic region, the same origins do not always fire simultaneously, confirming that different cells for the most part are replicating the same portion of the genome at different times (Figs. 2A, 3). To test if there is a more limited program of origin firing epigenetically inherited between consecutive S-phases, we determined if in successive S-phases individual origins that fire early in the first S-phase also fire early in the second. A synchronous culture was labeled with EdU, and origins firing in the next S-phase were labeled with BrdU (Supplemental Fig. S5A–C). Of 235 origins firing early in the second S-phase, 205 did not overlap with those firing early in the first. This indicates that there is no positive epigenetic memory of origin firing between successive S-phases. We conclude that there is not a strongly defined pattern of firing for the majority of origins as chromosomes proceed through replication.

There was also only limited overlap between the locations of clusters (Fig. 3). This can be seen by examination of four clusters (labeled A–D) identified on molecules replicated <20%. The position of these clusters was examined on other molecules replicated >20% (Fig. 3, gray vertical lines). It can be seen that they only partially overlap. The numbers of molecules that contained a cluster in the indicated positions were for A, 2/9; B, 3/10; C, 5/10; and D, 3/9. We conclude that clusters are not always formed on the same regions of the chromosomes, although it is possible that some regions do have a higher propensity to form clusters.

**Figure 4.**
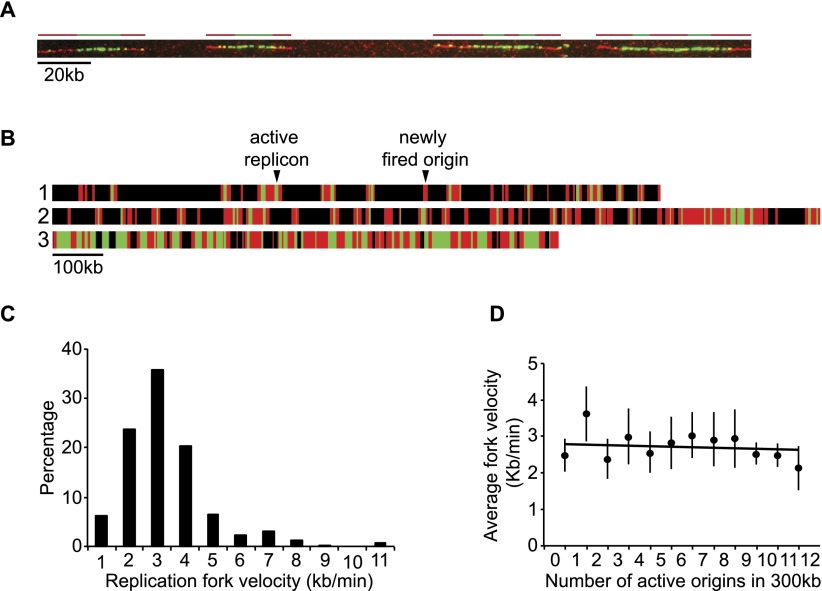
Fork velocities throughout S-phase. (*A*) Representative DNA molecule with six replicons. Green (BrdU pulse) and red (EdU chase) lines drawn on *top* of the molecules represent the position of six replicons. We measured the length of the red tracks immediately adjacent to green tracks that were flanked by unreplicated segments (progressive forks) and used the duration of the chase (3 min) to derive replication fork velocities. (*B*) Three representative single DNA molecules chosen from the population of molecules. Black bars represent unreplicated DNA, green bars represent the length and position of BrdU incorporation (pulse), and red bars represent the length and position of EdU incorporation (chase). An active replicon results from an origin fired during the BrdU pulse, which replication forks continue to incorporate during the EdU chase, and where at least one fork is flanked by an unreplicated segment at the end of the chase. A newly fired origin is an origin fired during the 3-min EdU chase (exclusively red tracks). (*C*) Histogram showing the distribution of 355 replication fork velocities. The average fork velocity is 2.8 kb/min. (*D*) The number of fired origins and the corresponding fork velocities were scored in consecutive 300-kb windows along single DNA molecules. Data points are represented as mean values ± SD.

The fact that the majority of origins fire in clusters raises the question as to whether replication proteins are accumulated in these clusters, which then result in faster rates of chain elongation. To test this possibility, fork velocity was measured by pulse labeling with BrdU, followed by a chase with EdU for 3 min, and measuring the track lengths of EdU that appear during its pulse time (Supplemental Fig. S3A; Fig. 4A,B). Most origins were bidirectional, and 80% of forks had velocities between 2 and 4 kb/min (Fig. 4B,C). The overall average fork velocity was 2.8 kb/min, similar to that measured using microarray approaches (Heichinger et al. 2006). To determine whether chain elongation was more rapid in clusters, we measured fork speed and plotted it against origin density. We found no correlation between fork velocity and origin densities ranging from one to two forks/300 kb to 10 to 12 forks/300 kb (Fig. 4D). We conclude that fork velocities in clusters (the high density regions) are similar to those located between clusters (low density regions).

We also examined the distribution of fired origins within the two ribosomal DNA (rDNA) loci, which consist of around 100 identical 10-kb sequence repeats, each of which contains a single origin. The cumulative frequencies of IODs can be described by two straight lines, with similar slopes to the rest of the genome (Supplemental Fig. S6). The average IOD within the rDNA loci was calculated from molecules <50% replicated as being 50 kb, meaning that approximately one in five of the potential origins is used. As with other chromosomal regions, there was no obvious overlap in individual origins or clusters within the rDNA locus that were firing in the different molecules (data not shown). All origins within rDNA loci are identical; but despite this, they fire stochastically, and clusters are formed at different locations, supporting the view that origin firing is generally not deterministic.

### Origin firing during S-phase

**Figure 5.**
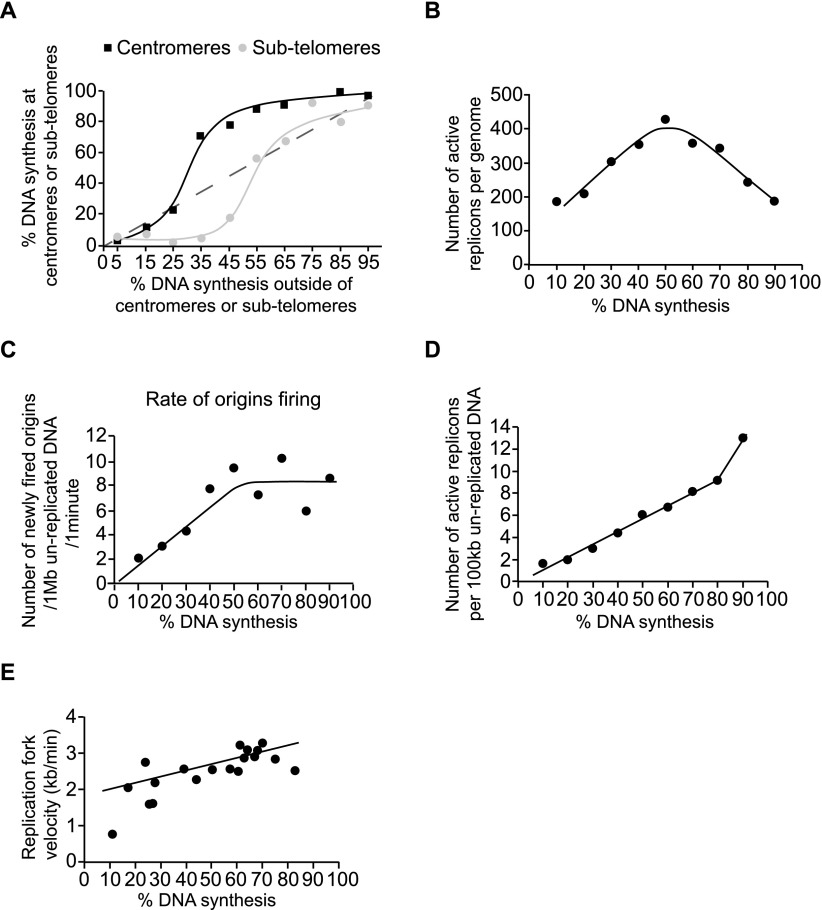
Origin firing throughout S-phase. (*A*) Centromeric and subtelomeric regions were identified on single DNA molecules relative to hybridized FISH probes in their vicinity. The extent of DNA synthesis for centromeres 1 and 3 and for subtelomeres on Chromosomes I and II are plotted as a function of the extent of DNA synthesis measured for the rest of each single DNA molecule. (*B*) The number of active replicons per genome was determined by analyzing pulse-/chase-labeled DNA molecules and is plotted as a function of the percentage of DNA synthesis on each molecule. (*C*) The number of newly fired origins during the 3-min chase with EdU is plotted as a function of the percentage of DNA synthesis for that DNA molecule. The rate of origin firing is expressed per minute per Mb of unreplicated DNA. (*D*) The number of active replicons per 100 kb of unreplicated DNA was determined from pulse-/chase-labeled DNA molecules and is plotted as a function of the percentage of DNA synthesis for each molecule. (*E*) The average replication fork velocity measured for each single DNA molecule is plotted as a function of the percentage of DNA synthesis on each molecule. For *A*–*E*, data points are represented as mean values.

In most of our experiments, we have analyzed single molecules averaging ∼2 Mb in length, each of which represents ∼15% of the genome. If the extent of DNA replication within these molecules reflects DNA replication in the rest of the genome within the cells from which they were derived, then analysis of these molecules would be informative about origin firing as cells proceed through S-phase. To test whether this assumption was reasonable, we examined molecules with centromeric regions and molecules with subtelomeric regions, which contain origins that replicate early and late in S-phase, respectively (Hayashi et al. 2009; Hayano et al. 2012; Tazumi et al. 2012). The extent of DNA replication in these regions was compared with the extent of replication in adjacent regions on the same molecules. Figure 5A shows that origins in centromeres replicate early (above the dashed line), and origins in subtelomeres replicate late (below the dashed line). We conclude that it is reasonable to use the extent of DNA replication of single molecules as a proxy for the extent of S-phase in the cells from which those molecules have been derived.

We analyzed single molecules to estimate the number of active replicons present in cells as they proceed through S-phase. We analyzed 30 DNA molecules pulse-labeled with BrdU (green) and chased with EdU (red) for 3 min (Fig. 4B). The short chase allowed identification of active replicons (origins with progressive forks in Fig. 4B) at the time of the chase, and we plotted the number of active replicons against the percentage of replication of the entire DNA molecule (Fig. 5B). The number of active replicons increased to around 350–400 per genome at the middle of S-phase and then decreased toward the completion of S-phase. We confirmed these data by analyzing another 167 BrdU-labeled single DNA molecules and determining the numbers of BrdU track ends as an estimate of fork numbers. These data are plotted in Supplemental Figure S7A against the percentage of DNA replication and show that the number of forks increases to 700–800 per genome in the middle of S-phase. Given that two forks are usually generated from a single origin, these figures also correspond to a maximum of 350–400 active replicons per genome at mid S-phase. We made an estimate of the total number of origins fired within a single S-phase by analyzing pulse/chased DNA molecules that had been nearly completely replicated (from 85% onward, for example molecule 3 on Fig. 4B). This gave an estimate of ∼500 origins or around half the total origins identified within the genome (Heichinger et al. 2006; Hayashi et al. 2007). However, this value of 500 origins is likely to be an underestimate, because in DNA molecules that are almost completely replicated, a number of tracks will result from merged replicons.

We also determined the rate of origin firing throughout S-phase by counting the number of newly fired origins during the 3-min chase with EdU (exclusively red tracks on Fig. 4B) and plotting it against the percentage of DNA replication on individual DNA molecules (Fig. 5C). The rate of origin firing increased from two newly fired origins per Mb per minute at the beginning of S-phase to eight newly fired origins per Mb per minute by mid S-phase, after which the rate of firing remained constant until the end of S-phase. We also plotted the number of active replicons per 100 kb of unreplicated DNA against the percentage of DNA replication of the entire molecule (Fig. 5D). The number of active replicons increased linearly from two per 100 kb of unreplicated DNA at the beginning of S-phase to around 10 per 100 kb of unreplicated DNA at the end of S-phase. We confirmed this result by determining the number of forks per 100 kb of unreplicated DNA by analyzing BrdU-labeled single DNA molecules and plotting this number against the percentage of DNA replication (Supplemental Fig. S7B). Similar to the pulse/chase experiment, the numbers gradually increased to around 20 forks per 100 kb of unreplicated DNA when cells reached 80% through S-phase, and then increased rapidly. Given these results, we estimate that toward the end of S-phase, 10 origins or more are being fired within a 100-kb unreplicated DNA region, corresponding to one origin firing at least every 10 kb. The accumulation of active replicons on unreplicated DNA toward the end of S-phase would contribute to completing genome replication. We also measured fork velocity as S-phase proceeded and found it to be relatively similar at between 2 and 3 kb/min, although it was somewhat lower in the first part of S-phase (Fig. 5E). We conclude that cells accumulate active replicons to a high level on unreplicated DNA, and that this assists the completion of genome replication at the end of S-phase.

### Replication in clusters and foci

Our results indicate that early in S-phase, origins fire stochastically along chromosomes, and then clusters of five or more origins appear, initially ∼100–200 kb in size but subsequently increasing in length (Fig. 2E; Supplemental Fig. S3B). This led us to examine if these clusters had any relationship to replication foci. Foci of replication factors such as PCNA have been identified in fission yeast (Meister et al. 2007), but foci of newly synthesized DNA have not been described. We used halogenated nucleotide labeling procedures to visualize DNA synthesis in situ. As cells proceeded through S-phase, more PCNA-GFP and BrdU foci appeared (Supplemental Fig. S8). Most BrdU foci colocalized with the PCNA-GFP foci. We conclude that BrdU foci represent loci where high levels of DNA synthesis are taking place and that they mostly colocalize with PCNA foci.

**Figure 6.**
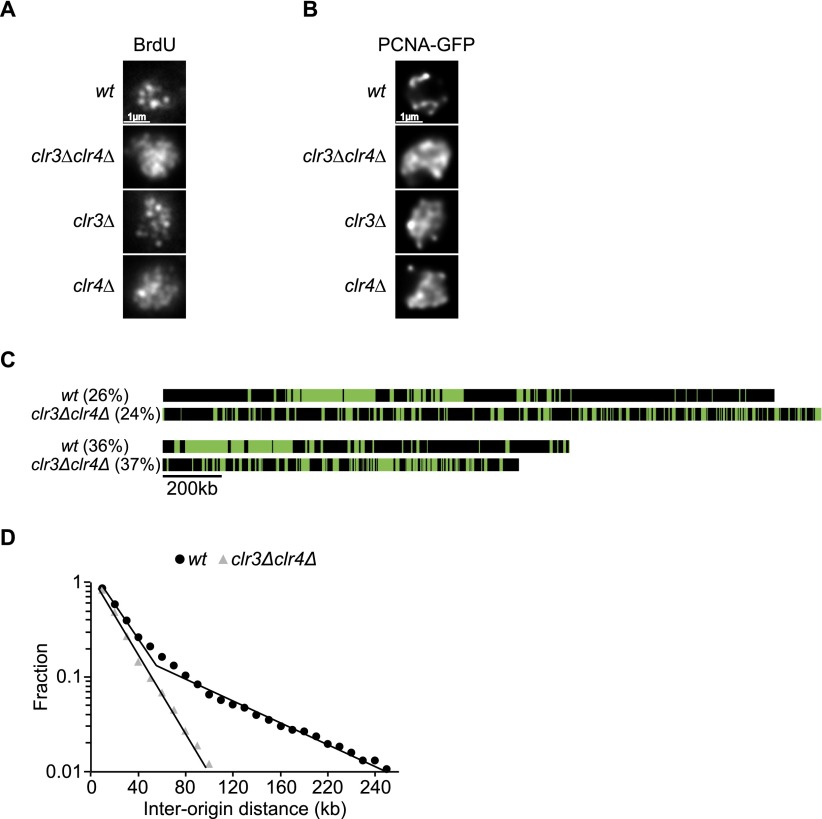
A *clr3∆clr4∆* mutant disrupts both the organization of replication foci within the nucleus and clusters with fired origins along the chromosomes. (*A*) Immunostaining of BrdU-labeled replication foci in *wt*, *clr3∆*, *clr4∆*, and *clr3∆clr4∆* cells. Images show an equatorial optical section. (*B*) Live imaging of PCNA-GFP-labeled replication foci. Images show an equatorial optical section. (*C*) Comparison between *wt* and *clr3∆clr4∆* molecules at the same percentage of DNA replication. Clusters are present in *wt* molecules but are not observed in *clr3∆clr4∆* molecules. (*D*) Semilog plot of the cumulative frequencies of IODs for molecules replicated from 20% to 50% for *wt* and *clr3∆clr4∆* cells. The values on the *y*-axis correspond to the fraction of IODs that are larger in size than the corresponding IOD on the *x*-axis. The data points for *clr3∆clr4∆* cells are fitted with a single straight line. The second straight line with the shallow slope described for *wt* cells is not present in *clr3∆clr4∆* cells.

To investigate if these foci are related to the clusters identified by our single molecule analyses, we tested candidate gene deletions to identify mutants that were disrupted in the organization of replication foci. We tested mutants affected in histone modifications (*hos2****∆***, *sir2****∆***, *clr3****∆***, and *clr4****∆***) and in chromosome organization (*swi6****∆***, *cds1****∆***, and *rad21-45*). The deletion of *clr4* (histone 3 lysine 9 methyltransferase) had the strongest effect, with the deletion of *clr3* (histone deacetylase) having a similar but weaker effect (Fig. 6A,B). A double mutant *clr3****∆****clr4****∆*** showed diffuse labeling with BrdU and PCNA-GFP, and hardly any foci were visible (Fig. 6A,B; Supplemental Fig. S9A,B). To examine if the alteration of foci organization was correlated with changes in origin distribution, we analyzed 18 molecules replicated between 20% and 50% from *clr3****∆****clr4****∆*** mutant cells (Fig. 6C). Figure 6D shows that the distribution of IODs was quite different in the *clr3****∆****clr4****∆*** cells compared with wild-type cells. The *clr3****∆****clr4****∆*** data points fit a single straight line with no inflexion and no indication of clustering. We conclude that the *clr3****∆****clr4****∆*** cells do not have well-defined clusters of fired origins, and that this is correlated with the absence of readily identifiable replication foci. This result is consistent with the idea that the basis of replication foci is clusters of fired origins. We also measured fork velocity in *clr3****∆****clr4****∆*** cells and found it to be reduced from the wild-type value of 2.75 kb/min to 1.65 kb/min (Supplemental Fig. S10). The number of active replicons was increased by 50%, compensating for the reduced fork velocity and resulting in a similar S-phase length for *wt* and *clr3****∆****clr4****∆*** cells (data not shown).

**Figure 7.**
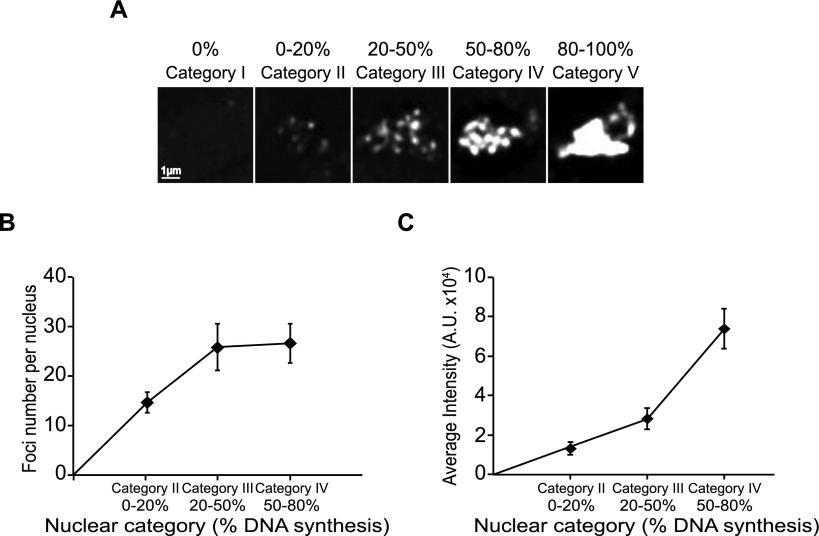
Characterization of replication foci during progression through S-phase. A synchronous cell culture was continuously labeled with 2 µM BrdU, and samples were taken at 10-min intervals starting just before the onset of S-phase and extending into G2. The sites with ongoing DNA synthesis were detected using immunofluorescence. (*A*) Five different categories of nuclei were created according to the percentage of replicated DNA: nuclei without detectable DNA synthesis (0%), nuclei replicated 0%–20%, 20%–50%, 50%–80%, and 80%–100% corresponding to categories I, II, III, IV, and V, respectively. (*B*) Replication foci number was scored for nuclei replicated from 0%–20%, 20%–50%, and 50%–80%. Foci number increased from 14 on average for nuclei replicated from 0%–20% to 26 on average for nuclei replicated from 20%–50%. For 50%–80% replicated nuclei, there are 27 foci on average, but replication foci appear more intense. Data are represented as mean values ± SD (*n* = 20). (*C*) Replication foci intensity increases throughout S-phase. The foci intensity increases 2.2-fold from nuclei 0%–20% replicated to nuclei 20%–50% replicated and an additional 2.6-fold to nuclei 50%–80% replicated. Data are represented as mean values ± SD (*n* = 20).

To relate foci to clusters during S-phase, we cataloged five different types of nuclei after BrdU labeling during a synchronized S-phase. We quantified the extent of BrdU incorporation into nuclei and identified nuclei that had carried out 80%–100% of maximal DNA replication as having essentially completed S-phase. Nuclei were then divided into different categories: category I at 0%; II at 0%–20%; III at 20%–50%; IV at 50%–80%; and V at 80%–100% of maximal DNA replication (Fig. 7A; Supplemental Fig. S11A). Foci were clearly visible in categories II, III, and IV. Cells proceeded through categories I–IV and accumulated in category V during S-phase progression (Supplemental Fig. S11B). We assessed foci number and total BrdU incorporation into individual foci in the different nuclei categories. Foci number per nucleus increased from 0 (category I) through 14 (category II), to 26–27 for categories III and IV (Fig. 7B). The intensity of foci also increased, rising 5.8-fold from category II to category IV (Fig. 7C). Assuming that categories II, III, and IV represent progression through S-phase of 0%–20%, 20%–50%, and 50%–80%, respectively, how do these numbers correspond to the numbers of clusters observed as cells proceed through S-phase? From the data in Supplemental Figure S12, we calculated that 5%–20% replicated molecules have 13 clusters per genome with eight origins on average per cluster, whereas the 20%–50% replicated molecules have 25 clusters and 12 origins on average per cluster. These results correlate with our estimate of foci number, as nuclei of category II have 14 foci and 5%–20% replicated molecules have 13 clusters, whereas nuclei of category III have 26 foci and 20%–50% replicated molecules have 25 clusters. This analysis supports the view that replication foci in nuclei correspond to the clusters observed on single DNA molecules.

## Discussion

We have analyzed the pattern of DNA synthesis along long single DNA molecules up to 5 Mb in length corresponding to large segments of fission yeast chromosomes to investigate the spatial and temporal organization of origin firing during a eukaryotic S-phase. Our spatial analyses indicate that at the beginning of S-phase, origins are activated sparsely throughout the chromosomes at random locations. About a quarter of the way into S-phase, clusters of more closely located fired origins appear embedded within the regions containing sparsely firing origins. These clusters are mostly 100–400 kb in length; and by mid S-phase, there are on average 25 clusters per genome, each containing 12 fired origins. Within the clusters, there was a 21-kb average IOD, whereas between clusters, it was about four times larger. In an earlier study (Patel et al. 2006), it was also reported that fired origins were randomly located, but no clusters were detected. We postulated that this might have been due to the shorter molecules of 200–500 kb that were analyzed. We tested this possibility by “cutting” in silico our longer molecules to pieces of 500 kb in length and showed that cumulative frequencies of the inter-replication track distances were essentially identical between our “cut” molecules and those from the study of Patel and colleagues, establishing that the earlier failure to detect clusters was due to the shorter molecules analyzed (data not shown). Stochastic origin firing on limited regions of the genome has also been reported for budding yeast, *Xenopus*, and mammalian cells (Czajkowsky et al. 2008; Labit et al. 2008; Demczuk et al. 2012; Saner et al. 2013).

Our analyses also indicated that for the majority of origins, there was no strong deterministic temporal program of origin firing. Most origins fired early in S-phase in one cell are not activated early in another cell. Even in successive cell cycles there is little overlap in the timing of origin firing, indicating that there is no epigenetic inheritance of the pattern of origin activation from one cell cycle to the next. Using a different method for DNA labeling, similar conclusions were reached by Patel and colleagues (Patel et al. 2006). There is also only limited overlap between the positions of the clusters that are formed as cells proceed through S-phase. However, a minority of origins is temporally organized. Approximately 20 origins, mostly near centromeres and mating type regions, have been shown to fire early in S-phase, whereas around 60 origins, mostly in subtelomeric regions, fire later (Heichinger et al. 2006; Hayashi et al. 2009; Hayano et al. 2012; Tazumi et al. 2012). Nine hundred and four origins have been mapped in the genome using microarrays with a 6.5-kb resolution (Heichinger et al. 2006), but our combing analysis with an improved 2-kb resolution has revealed that about one-third of origins are located within 6.5 kb of another origin and so would have been missed in the earlier analysis. Therefore, the total number of origins should be raised to around 1200, with an average IOD of ∼11 kb. These numbers indicate that the proportion of origins that are temporally organized is ∼7.5% of the total, and the majority of these are mapped at the heterochromatic loci. The remaining 92.5% fire more or less randomly. A consequence of most origins firing stochastically is that in different cells, different regions of the genome are replicated early or late during S-phase. It has been argued from analysis of mutation rates across budding yeast Chromosome VI that regions replicated late in S-phase have a higher mutation rate (Lang and Murray 2011). Stochastic origin firing throughout S-phase would also randomize which genome regions are replicated late in S-phase and so could spread potentially deleterious mutations more generally throughout the genome.

At the beginning of S-phase, the rate of origin firing is two origins per Mb per minute and increases gradually fourfold until just before mid S-phase, after which it remains constant. This results in the number of active replicons rising gradually to around 350–400 by mid S-phase, generating 700–800 active forks per genome. These results suggest that the initiation of S-phase is a rather gradual process, taking nearly half of S-phase before maximal origin activation is achieved. This could reflect either a progressive build-up of initiation factors or a gradual increase in chromatin accessibility or both. Such a scenario implies that the onset of S-phase should perhaps be viewed more of a progressive process rather than an abrupt transition. The number of active replicons and forks falls during the second half of S-phase, but the numbers of active replicons on DNA that have yet to be replicated increases more than 10-fold during S-phase. This results in the IODs of newly fired origins being ∼10 kb at the end of S-phase, which means that essentially all available origins are being fired at this late stage of DNA replication. Such a high density of origin firing will contribute to the completion of genome replication. These results support the proposal that limiting replication factors are increasingly concentrated on the reducing amounts of unreplicated DNA to assist the completion of genome replication (Rhind 2006; Goldar et al. 2009). Limiting factors previously proposed for replication include Cdc45, Sld2, Sld3, Dpb11, and the DDK protein kinase (Patel et al. 2008; Wu and Nurse 2009; Mantiero et al. 2011).

We have identified replication foci in the fission yeast nuclei that correspond to the previously identified PCNA-GFP foci (Meister et al. 2007). The replication foci are disrupted in *clr3****∆****clr4****∆*** cells altered in histone modifications, and clusters of fired origins are also not well defined in these cells. The simplest interpretation is that each cluster represents a focus, a conclusion supported by the observation that the numbers of foci observed correspond approximately to the numbers of clusters in the genome. Early in S-phase, there are 13 clusters per genome; and by mid S-phase there are 25 clusters, corresponding respectively to 14 foci and 26 foci. Fork velocity within the clusters is similar to the fork velocity in regions between clusters at 2.8 kb/min, so the concentration of replication factors in foci does not appear to increase the velocity of replication forks. The fact that alterations in histone modification change both origin clusters and replication foci suggests that chromatin structure plays a role in the control of origin firing as has been indicated by other studies (Vogelauer et al. 2002; Aggarwal and Calvi 2004; Knott et al. 2009). The persistence of regions of sparsely firing origins located between clusters requires maintenance of a normal chromatin structure, because in *clr3****∆****clr4****∆*** cells, origin firing is distributed more evenly throughout chromosomes. We could imagine that initially origins are fired randomly along chromosomes and that certain locations on the genome accumulate a higher density of fired origins, then a local positive feedback loop could induce further firing of adjacent origins to form a cluster. This could be a consequence of an opening up chromatin structure that allows easier local access of initiation factors (The ENCODE Project Consortium 2007; Karnani et al. 2010). In the *clr3****∆****clr4****∆*** cells, chromatin may already be more open, so the regions between clusters are not maintained. Alternatively, in a probabilistic fashion, segments of the chromosome could find themselves in regions of the nucleus where replication is more likely to be initiated leading to cluster formation. Nuclear compartments for replication and the stochastic association of large chromosomal regions with the nuclear envelope dependent on histone 3 lysine 9 methylation have been proposed from other studies (Labit et al. 2008; Kind et al. 2013).

Here we provide a genome-wide description of origin firing on long single DNA molecules representing extended regions of chromosomes as cells proceed through S-phase, the first for any eukaryote. Our data emphasize the importance of stochasticity in origin firing, compared with a more strictly deterministic program of origin firing. It also establishes that the organization of origin firing evolves during S-phase, with an initial random distribution throughout the chromosomes followed by the formation of clusters that form the basis of replication foci in nuclei. Given the similarities between origins in fission yeast and Metazoa, with a lack of a strict consensus sequence, AT richness, and a wide range of origin efficiencies, our observations concerning origin firing in fission yeast may also be relevant to origin firing in Metazoa. In particular, we suggest that there may previously have been too much emphasis on strictly deterministic models of origin firing during S-phase. In addition, the activation of increasing numbers of origins on unreplicated DNA toward the end of S-phase reported here could also have a role in Metazoan cells by assisting with the completion of genome duplication.

## Methods

### Strains and growth conditions

Standard fission yeast media and methods were used (Moreno et al. 1991). Strains are listed in Supplemental Table S1 and were generated by tetrad dissection. To obtain synchronized cell cultures, temperature sensitive *cdc25-22* strains were grown in minimal media (EMM) at 25°C to 1–2 × 10^6^ cells/mL, shifted to 36°C for 4 h to block cells in late G2, and then released at 25°C.

### DNA combing

Genomic DNA with ongoing DNA synthesis was prepared for combing in 1% low melting agarose Mb grade plugs (Bio-Rad) as described in (Fan et al. 1989). The plugs were extensively washed in TE 1X pH7.5, 100 mM NaCl, melted for 15 min at 70°C in MES 50 mM pH = 6.3, 100 mM NaCl, and incubated with 2 µL β Agarase (New England Biolabs) overnight at 42°C in a Teflon reservoir. The plug processing and gentle sample handling were responsible for maintaining the integrity of long DNA molecules in the presence of replication forks. Glass surfaces were activated in plasma cleaner (Harrick Plasma) and coated with 7-octenyltrichlorosilane in a gas chamber. Genomic DNA was combed on silanized glass surfaces using a combing machine (assembled with products from Thorlabs) at a speed of 900 µm/sec. After DNA denaturation, biotinylated DNA probes were hybridized to combed DNA in a humid chamber at 37°C, and probes were detected with streptavidin Alexa Fluor 594 (Invitrogen, S32356). To position and orient the combed DNA molecules on different chromosomes, sets of two probes for each chromosomal region were designed marking positions on average 1.2 Mb apart. Each chromosomal position is detected by a unique signature of two probes with differing lengths and distances between them. The positions on chromosomes marked by the probes are as follows: Chromosome I positions: 679–752 kb, 1151–1261 kb, 2420–2453 kb, 3922–4079 kb; Chromosome II positions: 785–852 kb, 2110–2120 kb (10.4kb *mat1-M*HindIII fragment of plasmid pDB262) (Beach 1983), 3214–3225 kb, 3712–3755 kb; and Chromosome III positions: 1139–1177 kb, and the rDNA probe (the 10.4kb repeat unit HindIII fragment of plasmid YIp10.4) (Toda et al. 1984). DNA was detected with mouse anti-single-stranded DNA antibody (Millipore, MAB3034), and Alexa Fluor 488 goat anti-mouse antibody (Invitrogen, A-11029), BrdU epitopes were detected with mouse anti-BrdU antibody (BD Biosciences, 347580) and DyLight 405 rat anti-mouse antibody (Jackson ImmunoResearch, 415-475-166) and DyLight 405 goat anti-rat antibody (Jackson ImmunoResearch, 112-475-143). For the pulse chase experiments, BrdU was detected with Alexa Fluor 488 mouse (clone MoBU-1) anti-BrdU antibody (Life Technologies, B35130) and Alexa Fluor 488 goat anti-mouse antibody (Invitrogen, A-11029), and EdU was detected using a Click-iT EdU Alexa Fluor Imaging Kit (Invitrogen, C10339). For each experiment, two controls were processed; to quantify the number of pixels corresponding to false negative staining, we used fully BrdU-labeled DNA; and to quantify the number of pixels corresponding to false positive staining, we used unlabeled DNA.

### Immunocytochemistry

Cells were fixed in 3% paraformaldehyde EM grade (Electron Microscopy Sciences) for 15 min at RT. Cells were washed three times in PEM (100 mM Pipes, 1 mM EGTA, 1 mM MgSO4 pH = 6.9) and once in PEMS (PEM plus 1.2 M Sorbitol). Cells were treated with 0.5 mg/mL Zymolyase-100T (Seikagaku) for 1 h at 37°C. Cells were permeabilized in PEMS plus 0.5% Triton X-100 and incubated in ice for 10 min. Cells were then washed in PEM and incubated in PEMBAL (PEM + 1% BSA, 100 mM Lysine hydrochloride, 0.1% NaN3, pH = 6.9) containing 5 mM MgSO4 for 30 min. BrdU epitopes were exposed by incubating cells for 2 h at 37°C in PEMBAL containing 5 mM MgSO4, nucleases from the 5-Bromo-2′-deoxy-uridine labeling and detection kit I (Roche), and mouse anti-BrdU antibody (BD Biosciences, 347580). Cells were then washed three times with PEMBAL and incubated overnight with mouse anti-BrdU antibody (BD Biosciences, 347580). Cells were then washed three times in PEMBAL, incubated with Alexa Fluor 488 goat anti-mouse antibody (Invitrogen, A-11029) for 3 h, washed in PEMBAL, in PBS, and stained with DAPI. Alternatively, for heat denaturation, after permeabilization, cells were recovered in PEMS and incubated for 10 min at 95°C, followed by incubation in an ice/H2O bath for 5 min.

### Imaging and quantification

Images for cells and DNA molecules were collected in Metamorph (MDS Analytical Technologies) using an epifluorescence microscope (Axioplan 2, Carl Zeiss, Inc.) equipped with a Zeiss Plan-FLUAR 63×/1.40 or 100×/1.45 lenses (Carl Zeiss, Inc.) and CoolSNAP HQ camera (Roper Scientific). Images were deconvolved using Huygens software (Scientific Volume Imaging) at fast mode and 50 iterations. Alternatively, cells were imaged with a DeltaVision image restoration microscope system (Applied Precision) mounted on an Olympus IX-70 microscope and fitted with a 100×/1,40 UPLSAPO objective lens and CoolSNAP QE CCD camera (Photometrics). Images were deconvolved with SoftWorx (Applied Precision) and exported to MetaMorph (Universal Imaging) for analysis and quantification. Integrated intensity was determined after applying a threshold determined from unlabeled cells to remove background. The quantification of the signal was made by summing the integrated pixel intensities within the whole nucleus and normalized to the fully labeled nuclei, which were considered 100% replicated.

## Supplementary Material

Supplemental Material
