# Supplementary material for: The spatial and temporal organization of origin firing during the S-phase of fission yeast
Source: Genome Res. 2015 Mar;25(3):391–401. doi: 10.1101/gr.180372.114 (PMC4352884; doi:10.1101/gr.180372.114)
Supplement: Supplemental Material [file supp_25_3_391__index.html]

The spatial and temporal organization of origin firing during the S-phase of fission yeast — The spatial and temporal organization of origin firing during the S-phase of fission yeast — Supplemental Material 

# The spatial and temporal organization of origin firing during the S-phase of fission yeast

## Supplemental Material

**Files in this Data Supplement:**

- Supplemental Material.pdf
- Table\_S1.ai
- Table\_S2.xlsx
