# Supplementary material for: The spatial and temporal organization of origin firing during the S-phase of fission yeast
Source: Genome Res. 2015 Mar;25(3):391–401. doi: 10.1101/gr.180372.114 (PMC4352884; doi:10.1101/gr.180372.114)
Supplement: Supplemental Material [file supp_gr.180372.114_Supplemental_Material.pdf]

Supplemental Material

The spatial and temporal organisation of origin firing during the S-phase of fission yeast

Atanas Kaykov and Paul Nurse

Figures S1-S15
Tables S1 and S2
Supplemental References

Kaykov_Figure S1

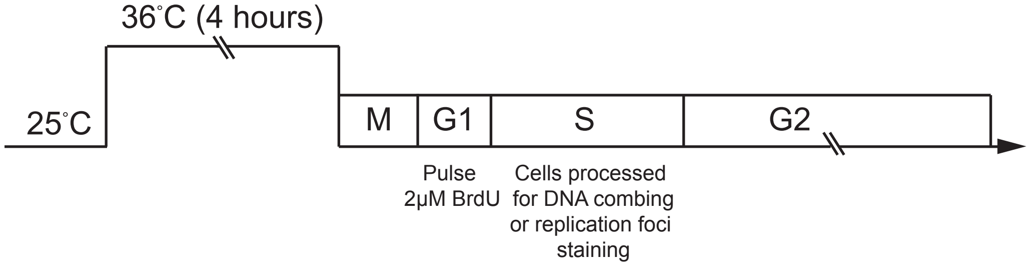

Figure S1. Scheme for labelling fission yeast cells

Cells were synchronized for entry into S-phase by blocking *cdc25ts* cells in G2 at 36˚C followed by a shift down to 25˚C, after which they proceeded synchronously through mitosis and a short G1 into S-phase. 2μM BrdU was added to a synchronized culture in G1 allowing cells to enter S-phase in the presence of BrdU. DNA was extracted for combing from two different time points in S-phase; 65 minutes after release at 25˚C (corresponding to 20% DNA replication for the population, estimated from FACS profile) and 75 minutes (corresponding to 50% of DNA replication for the population, estimated from FACS profile). Alternatively, cells were taken at 10 minute intervals throughout S-phase, fixed using PFA, and replication foci were immunodetected.

Kaykov_Figure S2

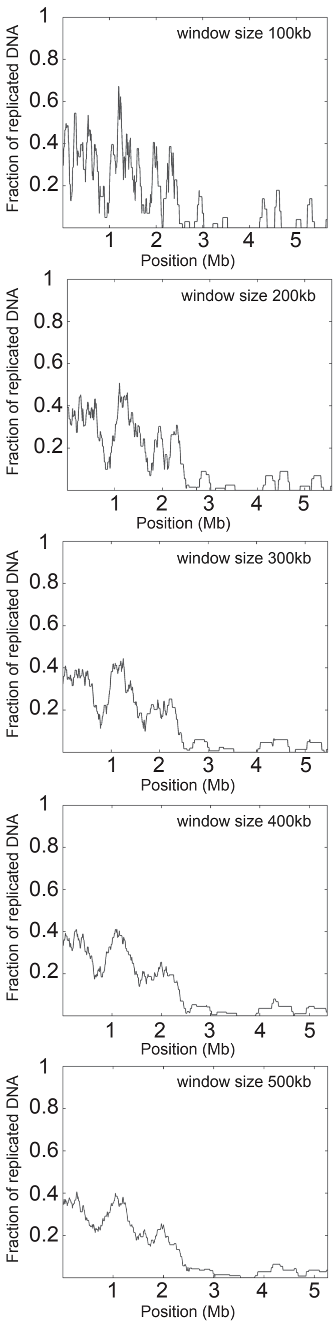

Figure S2. Moving average analysis of DNA replication along single DNA molecules

The 13% replicated 5.6Mb DNA molecule (shown in Figure 1A) was analysed with 5 different window sizes (100kb, 200kb, 300kb, 400kb and 500kb) with a step size of 2kb (the resolution of the combing technique). The profiles generated using the 200kb window size showed 4 significant peaks, located on the left half of the molecule representing chromosomal domains rich in newly synthesized DNA. Poorly replicated DNA regions were located between them. The right half of the molecule was poor in newly synthesized DNA and significant peaks could not be identified. The profiles generated with a 100kb window size were noisy, with many narrow peaks with rather high levels of DNA replication between them, suggesting that this window size is smaller than the clusters size. On the other hand the 4 significant peaks were less well defined using 300-500kb windows sizes with increasingly higher levels of DNA replication between them suggesting that these window sizes are larger than the cluster size.

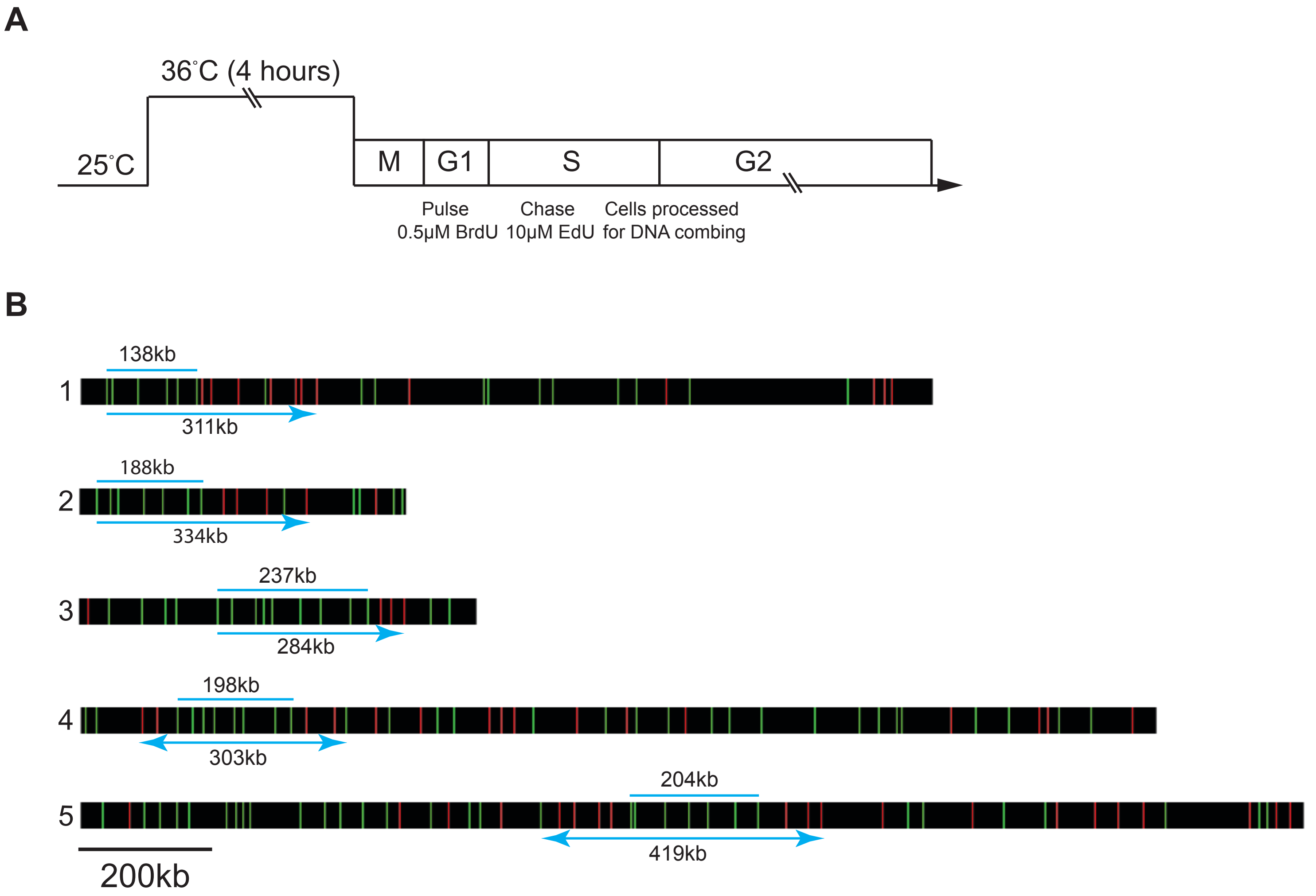

Figure S3. Cluster size increases as cells proceed through S-phase

(*A*) Scheme for labelling fission yeast cells. Cells were synchronized for entry into S-phase by blocking *cdc25ts* cells in G2 at 36˚C followed by a shift down to 25˚C, after which they proceeded synchronously through mitosis and a short G1 into S-phase. 0.5μM BrdU was added to a synchronous culture in G1 and at 67 minutes after release (early S-phase) the label was chased with 10μM EdU for 9 minutes (corresponding to 3 minutes actual incorporation allowing for the lag in uptake). DNA was extracted for combing from cells 75 minutes after release (corresponding to 50% of DNA replication for the population, estimated from FACS profile).

(*B*) A synchronous culture entered S-phase incorporating BrdU (added to the media in G1), and at mid S-phase BrdU was chased with EdU for 3 minutes. Replication origins fired during the time of the BrdU pulse are shown with green bars and origins fired exclusively during the EdU chase are shown with red bars. Five representative single DNA molecules are shown. Clusters of 5 or more fired origins during the time of the BrdU pulse, located no more than 40kb apart are indicated with a blue line on top of each DNA molecule. The size of each cluster is indicated. Origins firing during the EdU chase within 40kb of the edges of previously formed clusters increase cluster size and blue arrows below each DNA molecule show the directionality of growth for each cluster. Cluster sizes after EdU chase are indicated below each blue arrow. We scored 8 (57%) clusters that became extended during the time of the chase and 6 (43%) clusters that did not.

Kaykov_Figure S4

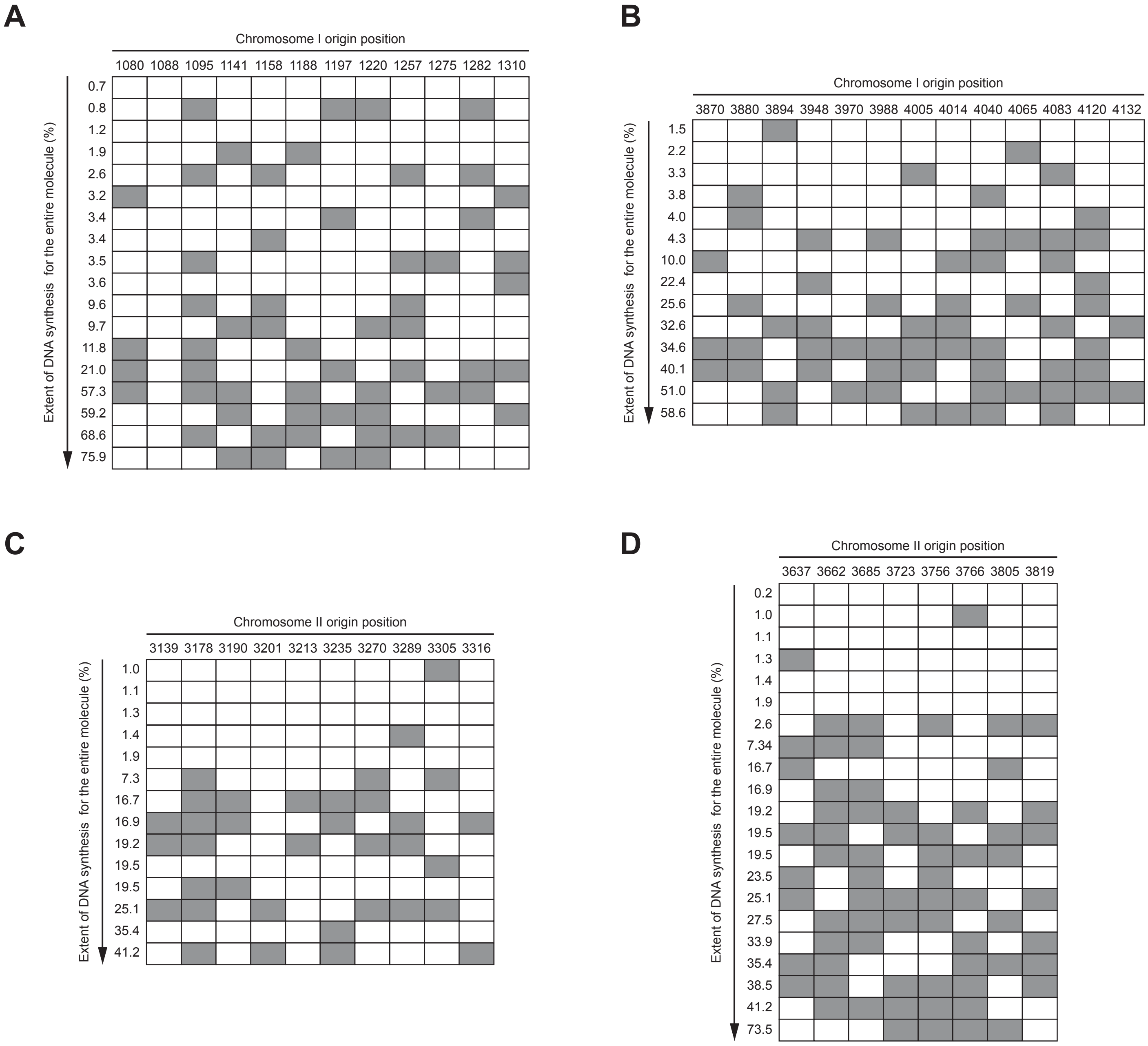

Figure S4. Individual origins fire randomly

(*A-D*) Individual origins were mapped across four different regions of the genome on single DNA molecules. Each panel represents a region identified by hybridized FISH probes in the middle of the region with additional 70kb from either side of the probe; panels A and B represent two regions from chromosome I located from 1060kb to 1320kb and from 3860kb to 4150kb, respectively; panels C and D represent two regions from chromosome II located from 3130kb to 3320kb and from 3630kb to 3820kb, respectively. The position of individual origins is shown on top of each panel. The extent of DNA replication for each molecule is shown on the left side of the panel. The white squares represent un-fired origins while grey squares represent fired origins.

Kaykov_Figure S5

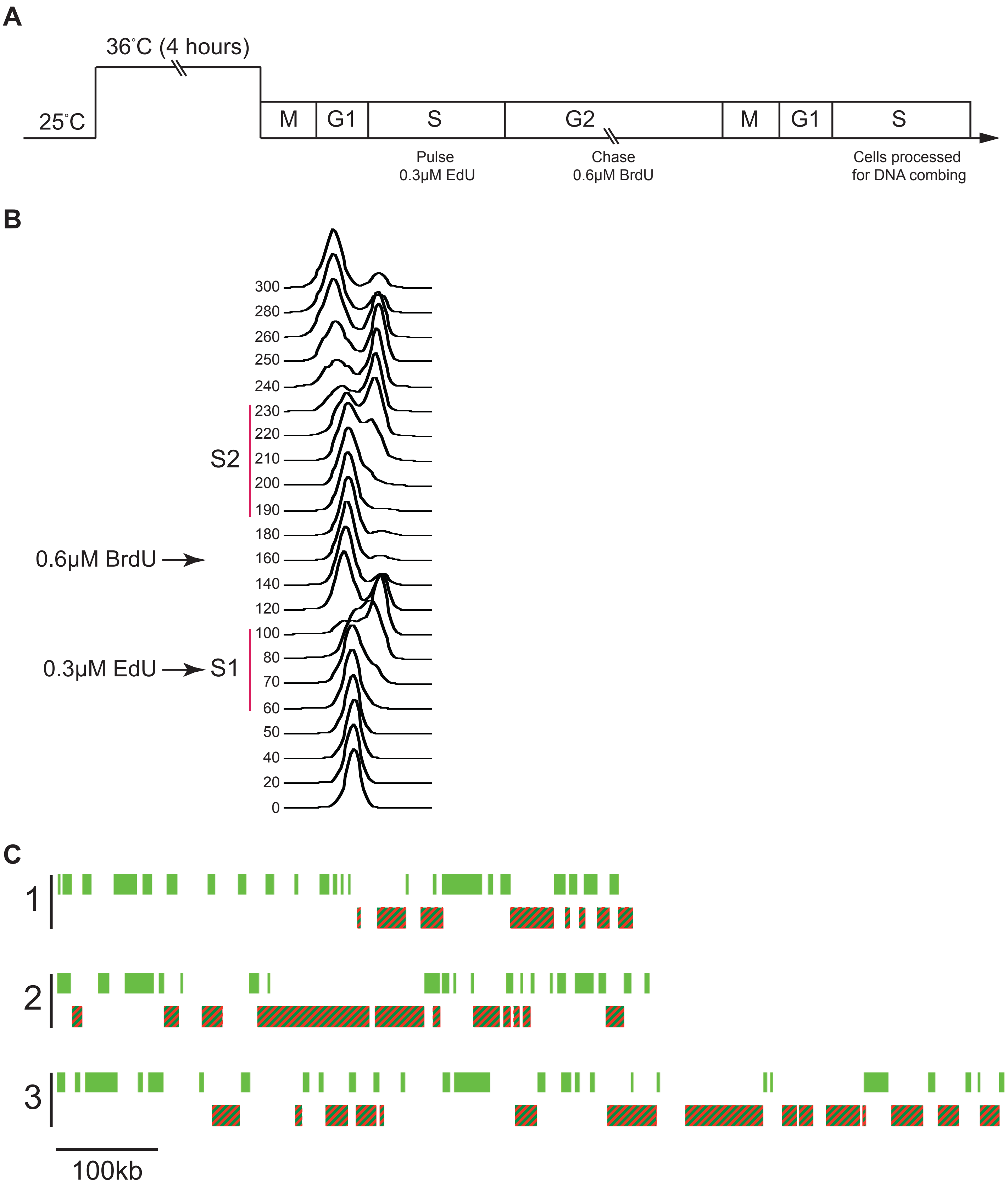

Figure S5. Origin firing in consecutive S-phases

(A) Scheme for differential labelling of fired origins during two consecutive S-phases. Cells were synchronized for entry into S-phase by blocking *cdc25ts* cells in G2 at 36°C followed by a shift down to 25°C, after which they proceeded synchronously through mitosis and a short G1 into S-phase. 0.3μM EdU was added to the culture at mid-S-phase (S1) and the label was chased with 0.6μM BrdU at mid-G2 phase. Cells proceeded synchronously through G2, second M and G1 phases, and entered a second S-phase (S2) in the presence of 0.6μM BrdU. DNA was extracted for combing from cells arrested at the beginning of S2 (210 minutes after release, corresponding to 30% DNA replication for the population, estimated from FACS profile).

(B) FACS profile of synchronous *cdc25ts* cell population. The numbers on the left side of the FACS profile indicate the time points after release from temperature block. The first S-phase (S1) and the consecutive S-phase (S2) are indicated with red lines. The time of EdU pulse in mid-S1 and the time of BrdU chase in mid-G2 are indicated with arrows.

(C) Origin firing in consecutive S-phases. A synchronous culture was labelled at mid S-phase with 0.3μM EdU and was allowed to complete DNA replication (early replicating regions corresponding to unlabeled DNA are represented with green bars). Before the onset of the next S-phase, the media was filtered and cells were incubated in pre-conditioned media with 0.6μM BrdU, allowing cells to enter the second S-phase incorporating BrdU. However, BrdU was incorporated with EdU in S2 (early replicating regions in S2, corresponding to BrdU and EdU co-labeling are represented with bars with green and red stripes). This allowed imaging of differentially labelled replication origins fired during two consecutive S-phases on the same DNA molecule. The data indicate that there is no strong positive correlation between early replicated regions in sequential S-phases (origins used in early S1 are not used in early S2). The data cannot provide insight beyond this due to the technical limitations associated with the co-incorporation of BrdU and EdU during the chase. Three representative single molecules are shown.

Kaykov_Figure S6

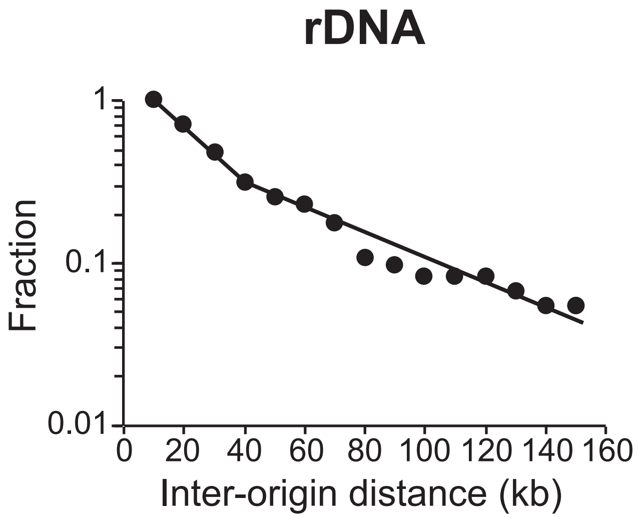

Figure S6. Origin firing in ribosomal DNA loci

Semi-log plot of the cumulative frequencies of IODs of fired origins in rDNA loci. Two straight lines with different slopes can be fitted on the data points reflecting two different regimes of stochastic origin firing operating in the rDNA loci. Despite the fact that rDNA loci consist of approximately one hundred identical 10Kb sequence repeats, each containing an origin, origins fire in clusters with similar characteristics to the clusters mapped in the rest of the genome.

Kaykov_Figure S7

A

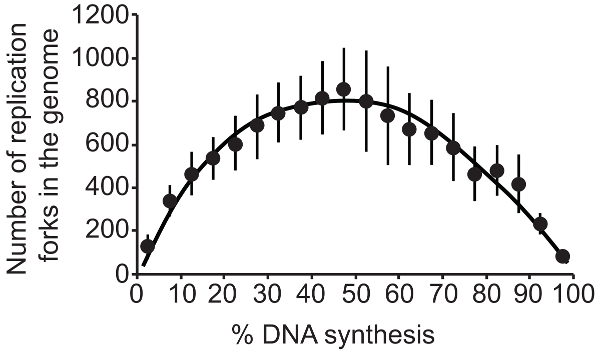

B

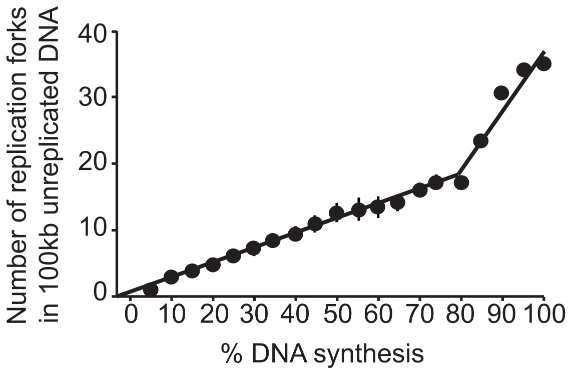

Figure S7. Origin firing throughout S-phase determined from BrdU pulse labelling

(*A*) Replication track ends corresponding to replication forks were counted on BrdU pulse labelled DNA molecules and the percentage of DNA replication for each molecule was determined. The number of replication forks was normalized to the entire genome and plotted as a function of the percentage of DNA replication. Data are represented as mean values ± SD.

(*B*) The number of replication forks per 100kb of un-replicated DNA is plotted as a function of the percentage of DNA replication for each molecule. Data are represented as mean values ± SD.

Kaykov_Figure S8

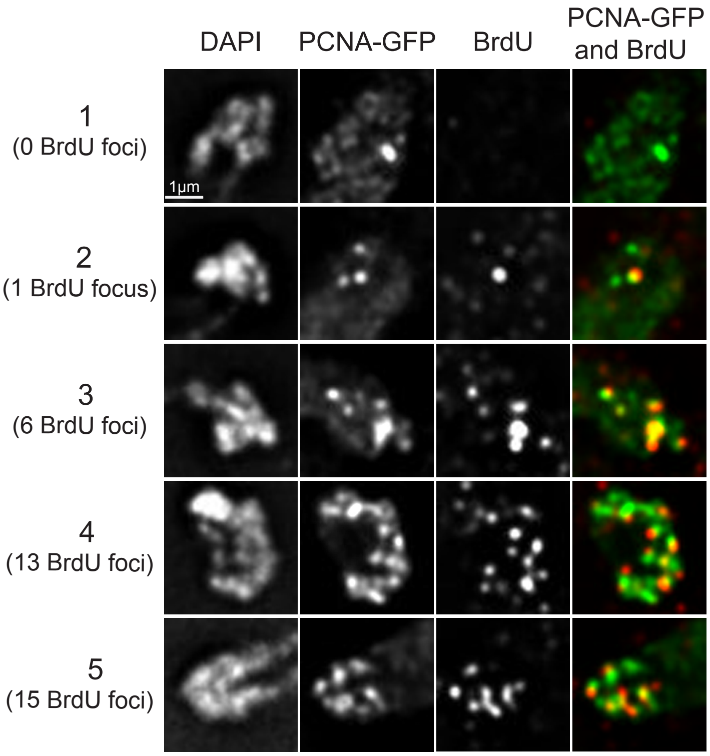

Figure S8. PCNA-GFP foci correspond to BrdU foci

Cells expressing PCNA-GFP were synchronized using *cdc25ts* and BrdU pulse labelled at the beginning of S-phase. A single focal plane imaged in three different channels corresponding to DAPI, PCNA-GFP and BrdU signals is shown. The nuclei are ordered according to their progression through S-phase, derived from the number and intensity of BrdU replication foci present in each nucleus. Nucleus 1 was at the beginning of S-phase since one PCNA-GFP focus had formed but DNA synthesis was not detected as measured by BrdU incorporation. Nucleus 2 had formed 3 PCNA-GFP foci but only one co-localized with the single BrdU focus. In nuclei 3, 4 and 5 replication foci number increased to 6, 13 and 15 respectively.

Kaykov_Figure S9

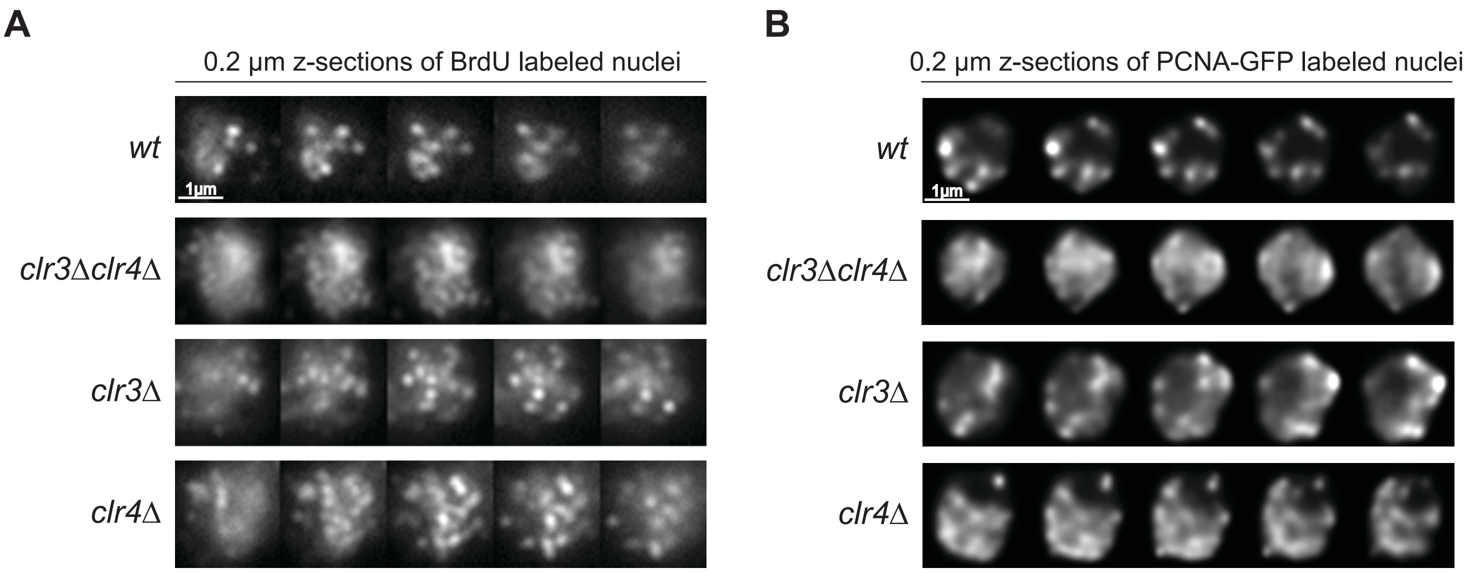

Figure S9. Spatial organisation of BrdU labelled, and PCNA-GFP replication foci in *wt*, *clr3*Δ, *clr4*Δ, and *clr3*Δ*clr4*Δ cells

(*A*) An exponentially growing culture was pulse labelled for 5 minutes with BrdU. After a short PFA fixation, sites of ongoing DNA synthesis were detected using immunofluorescence. Replication foci were imaged in septated cells (cells undergoing S-phase). Five consecutive optical sections (spacing 0.2 μm) of a representative nucleus for each strain are shown.

(*B*) Replication foci were imaged in live septated cells (cells undergoing S-phase). A deconvolved stack of five consecutive optical sections (spacing 0.2 μm) of a representative nucleus for each strain is shown.

Kaykov_Figure S10

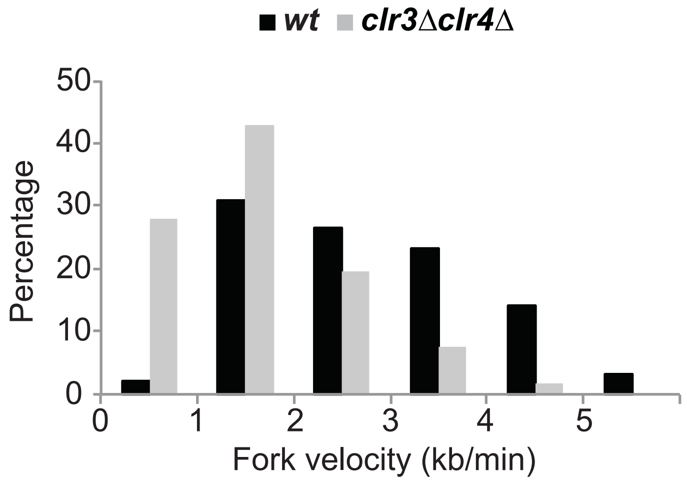

Figure S10. Replication fork velocity in *clr3Δclr4Δ* cells

Replication fork velocity was measured in the same manner as in Figure 4 for *clr3Δclr4Δ* and *wt* cells. The average *wt* fork velocity was 2.75kb/minute whereas *clr3Δclr4Δ* cells had a reduced fork velocity of 1.65kb/minute.

Kaykov_Figure S11

A

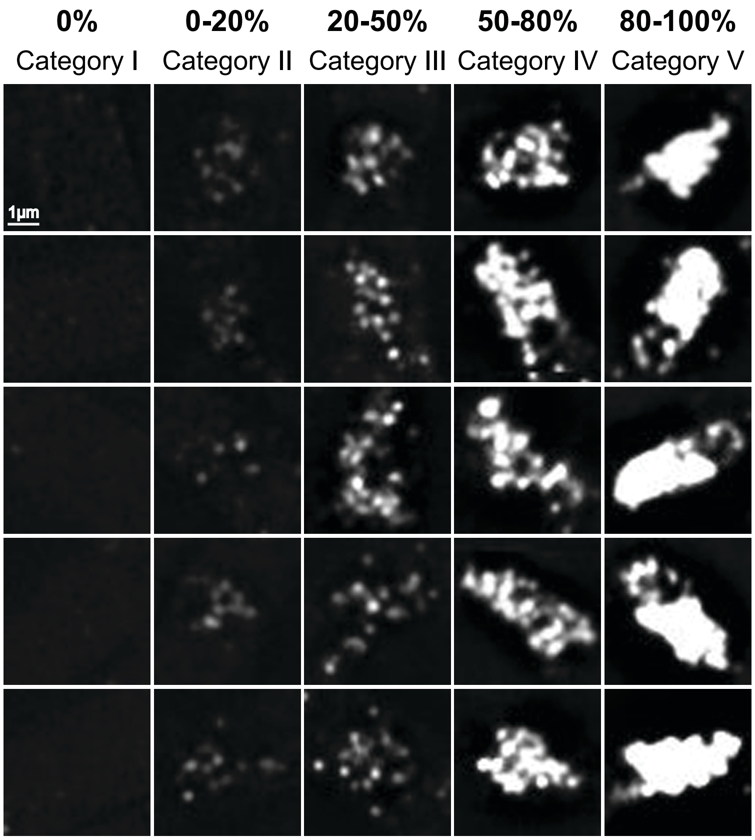

B

Time after release (min)	Percentage of each nuclei category					number scored nuclei
	category I	category II	category III	category IV	category V	
60	61	13	16	10	-	271
70	46	13	18	20	3	310
80	15	10	19	32	24	320
90	6	3	10	32	49	364
100	4	-	6	35	55	286
110	3	-	1	28	68	269
120	1	-	-	11	88	267

Kaykov_Figure S11

Figure S11. Fission yeast form BrdU replication foci during progression through S-phase

A synchronous cell culture was continuously labelled with 2μM BrdU and samples were taken at 10 minute intervals starting just before the onset of S-phase. Cells were PFA fixed and sites of ongoing DNA synthesis were detected using immunofluorescence.

(*A*) Five different categories of nuclei were created according to the percentage of synthesised DNA; nuclei without detectable DNA replication 0%, nuclei replicated from 0-20%, 20-50%, 50-80% and 80-100% corresponding to categories I, II, III, IV and V respectively. Five examples of each category are shown.

(*B*) The percentage of nuclei corresponding to each category was scored for each time point. Sixty minutes after release from temperature block, the population contained mainly nuclei that had not yet synthesised detectable amounts of DNA (61%). At the end of the time course, 120 minutes after release, 88% of the nuclei were about to complete S-phase.

Kaykov_Figure S12

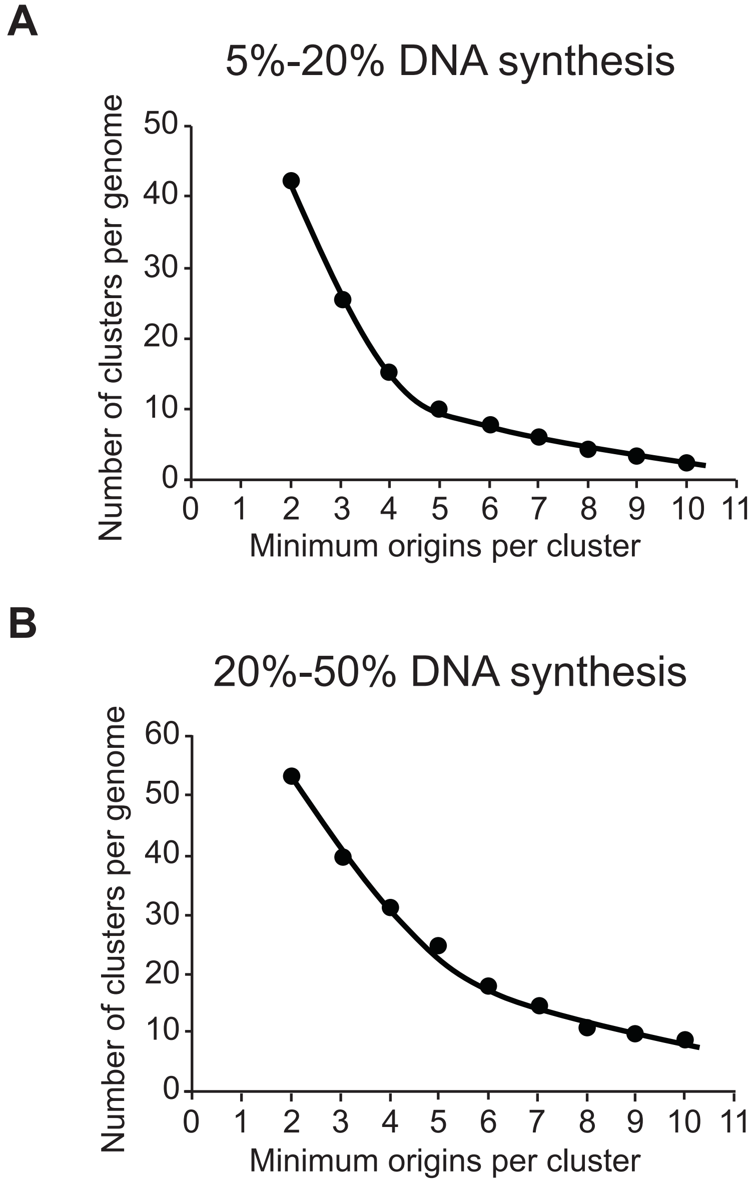

Figure S12. Estimate of the minimum number of origins within clusters

Plot of the minimum number of origins fired within 40kb of each other for molecules replicated from 5% to 20% (A) or 20% to 50% (B). The curves can be decomposed into two straight lines which intersect between 4 to 5 origins per clusters for (A) or 5 to 6 origins per cluster for (B). Assuming that a minimum of 5 fired origins define a cluster, cells having replicated 20% or 50% of their DNA will have on average 13 or 25 clusters, respectively.

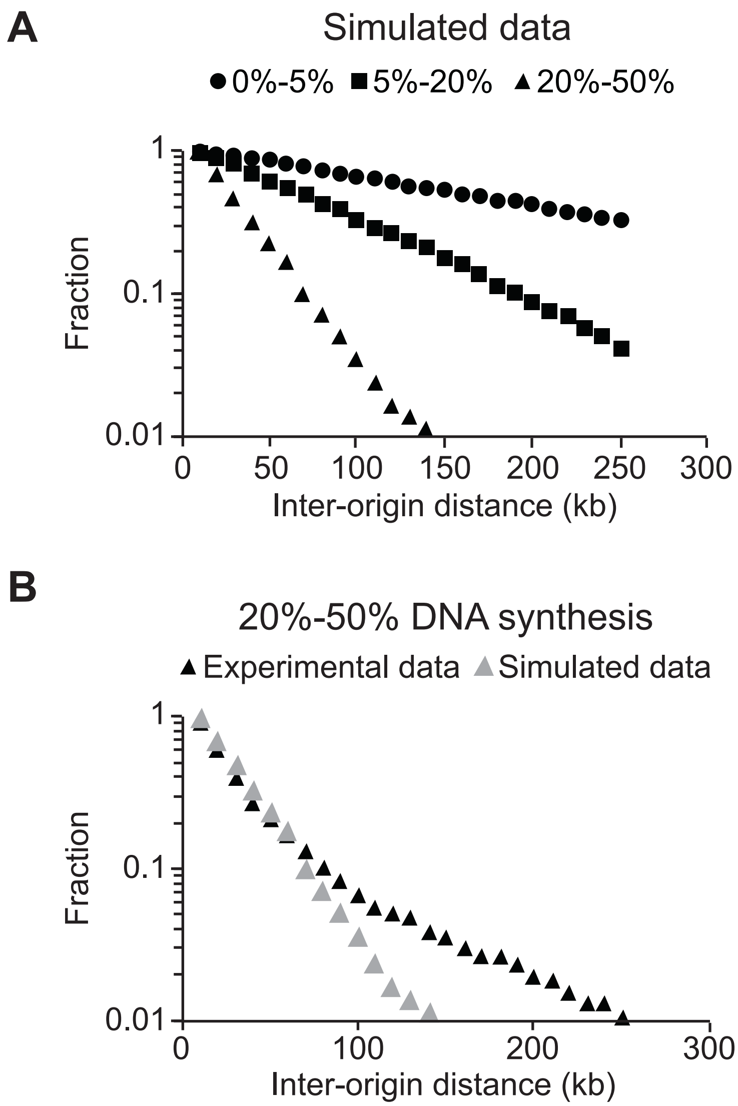

Figure S13. Distribution of IODs assuming purely stochastic origin firing

We simulated the process of DNA replication for fission yeast assuming purely stochastic origin firing on un-replicated genomic loci (excluding already replicated DNA). Each origin generates bidirectional replication forks with an average velocity of 2.8Kb/minute and rates of origin firing of 1, 2 and 4 newly fired origins/minute/Mb for 5%, 20% and 50% advancement of DNA replication respectively (these parameters were measured experimentally in this study Figures 4C and 5C).

(A) Semi-log plot of the cumulative frequencies of IODs assuming stochastic origin firing for molecules sampled according to their extent of DNA synthesis; black circles, black squares and black triangles correspond to molecules replicated from 0-5%, 5-20% and 20-50%, respectively. The values on the y-axis correspond to the fraction of IODs that are larger in size than the corresponding IOD on the x-axis. Origin firing is random as the cumulative frequencies of IODs fit straight lines for different extent of DNA replication.

(B) Semi-log plot of the cumulative frequency of IODs for molecules replicated from 20% to 50% for experimental data and simulated data represented as black and grey triangles respectively. The experimental measurements deviate significantly from the purely stochastic prediction demonstrating that origin firing is not random.

Kaykov_Figure S14

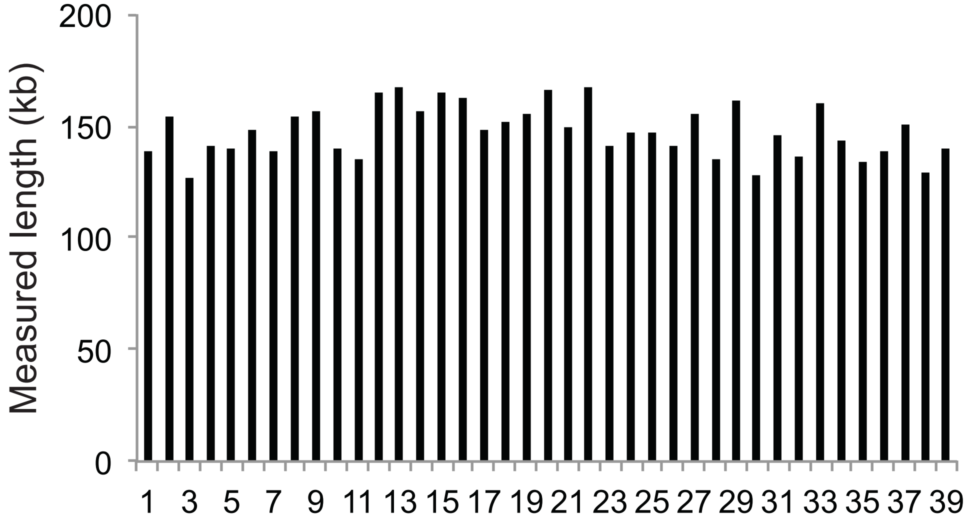

Figure S14. Histogram of the measured length of the hybridised 150kb FISH probe

The average length is 146kb and the standard deviation is 15kb.

A

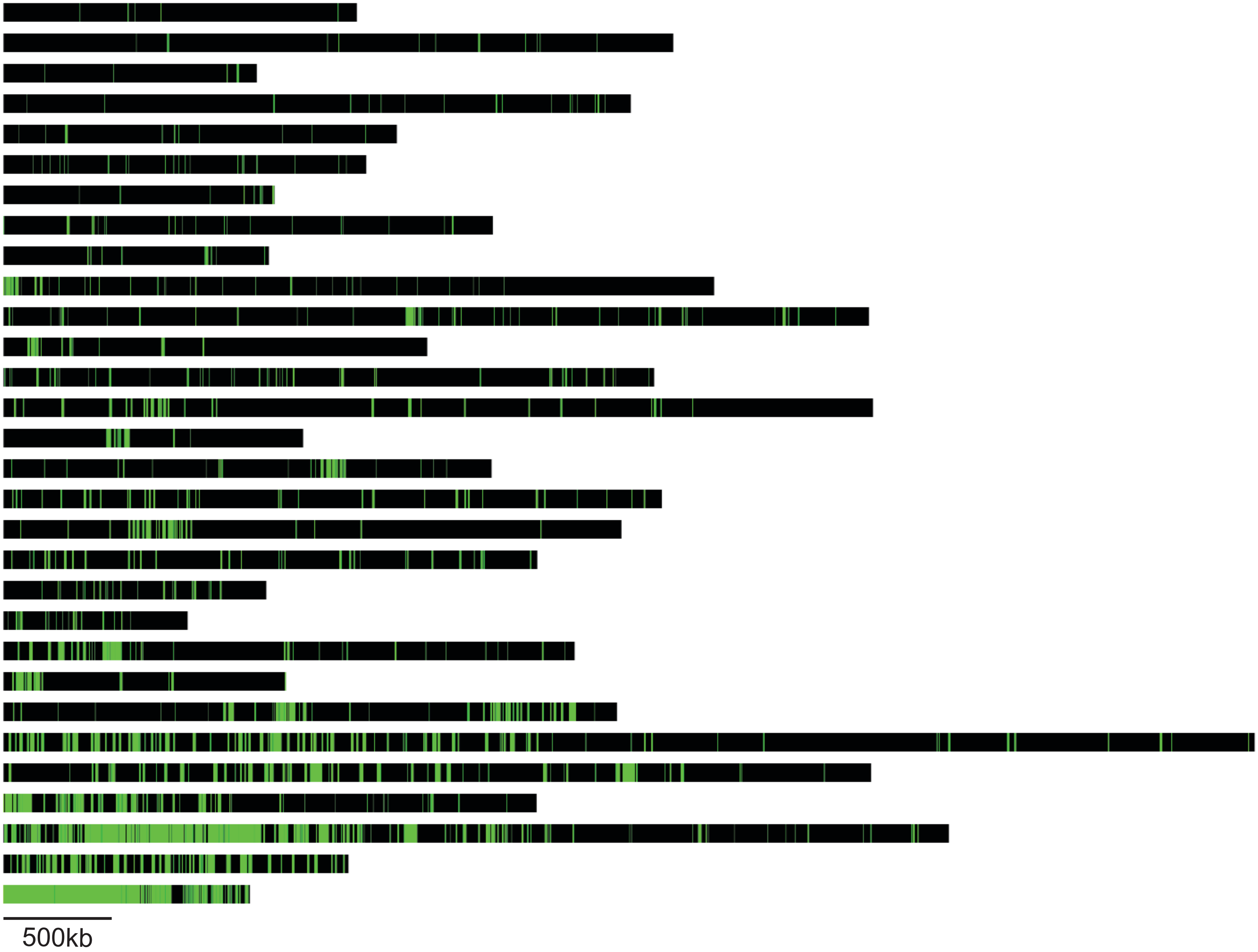

B

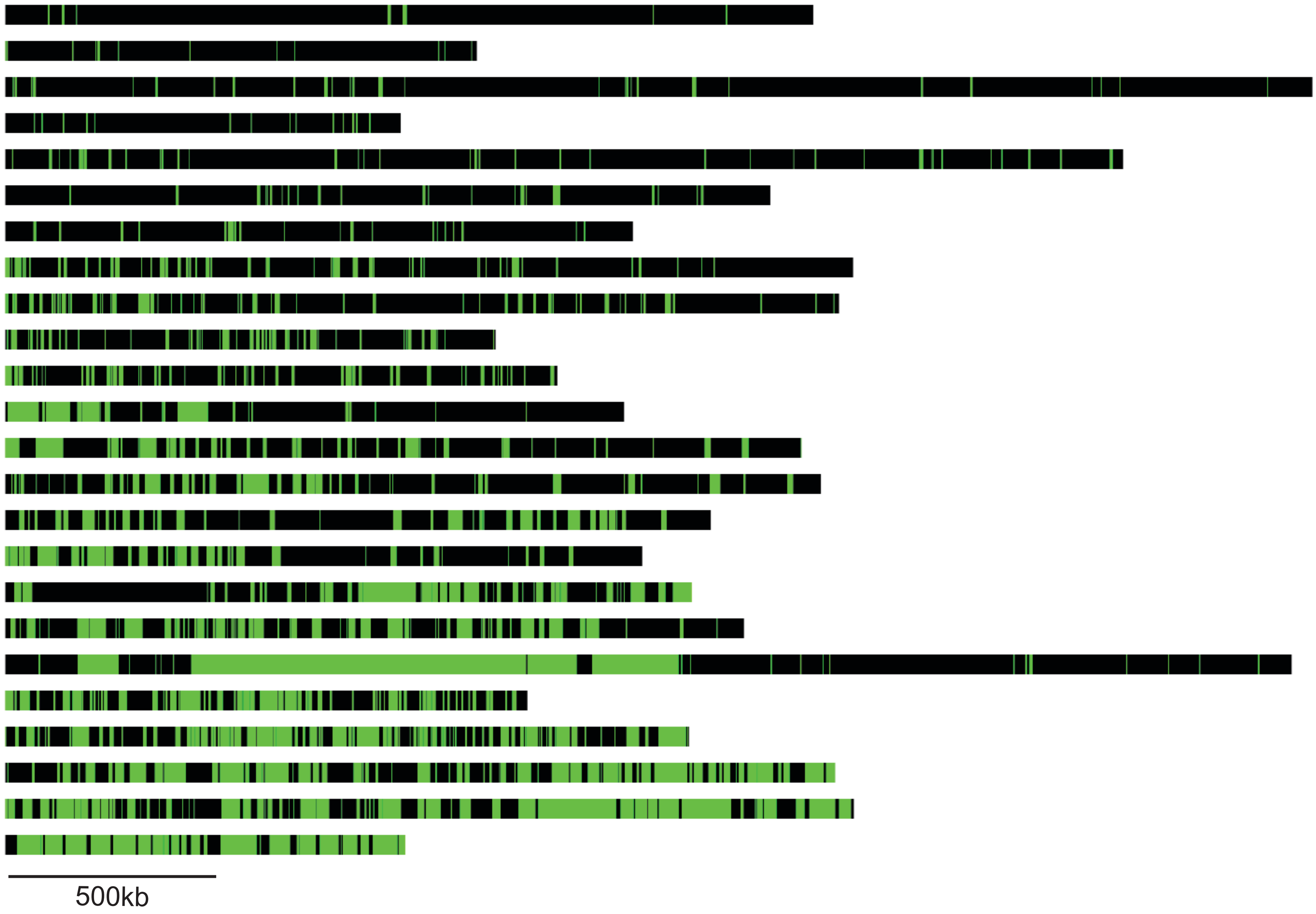

Kaykov_Figure S15

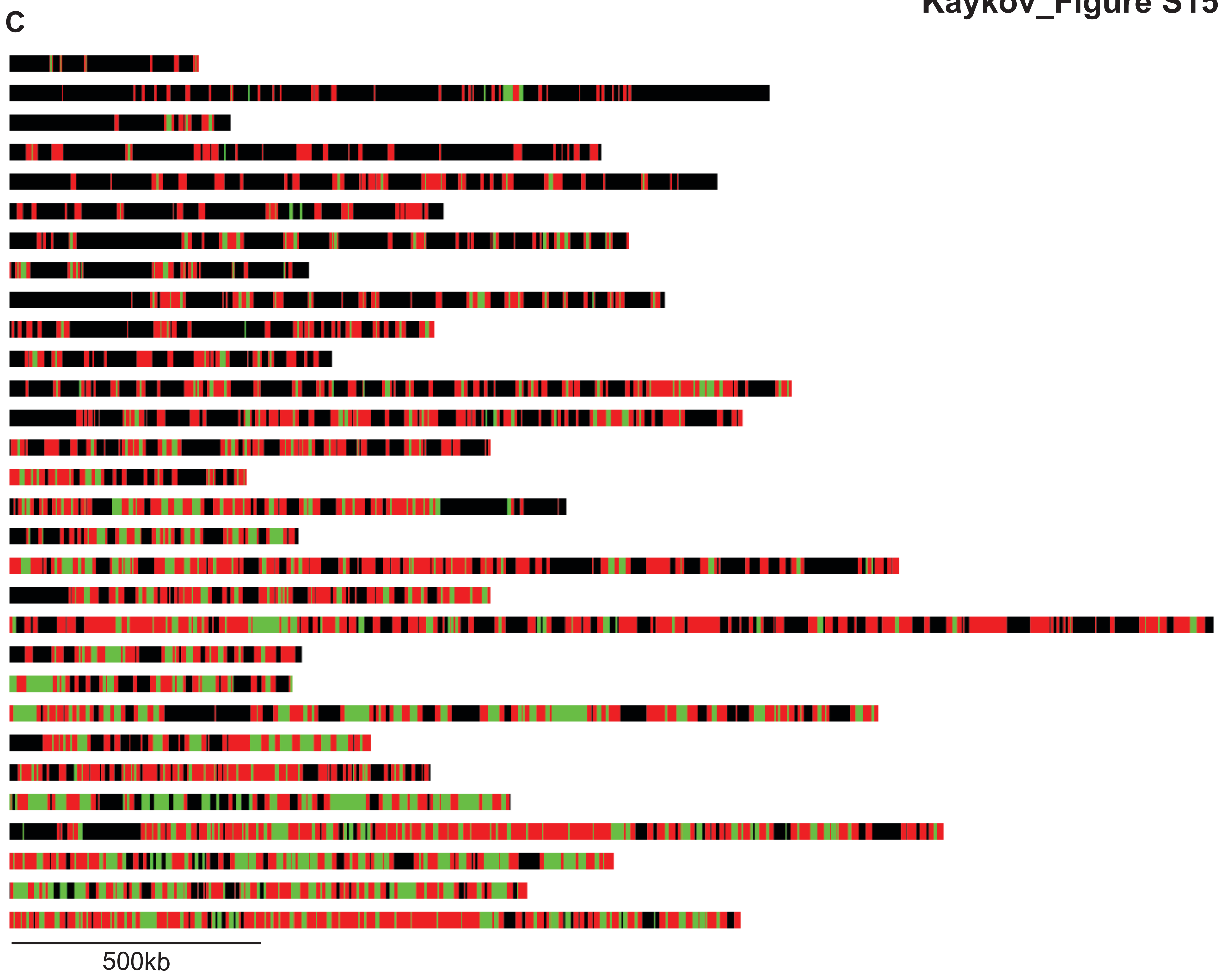

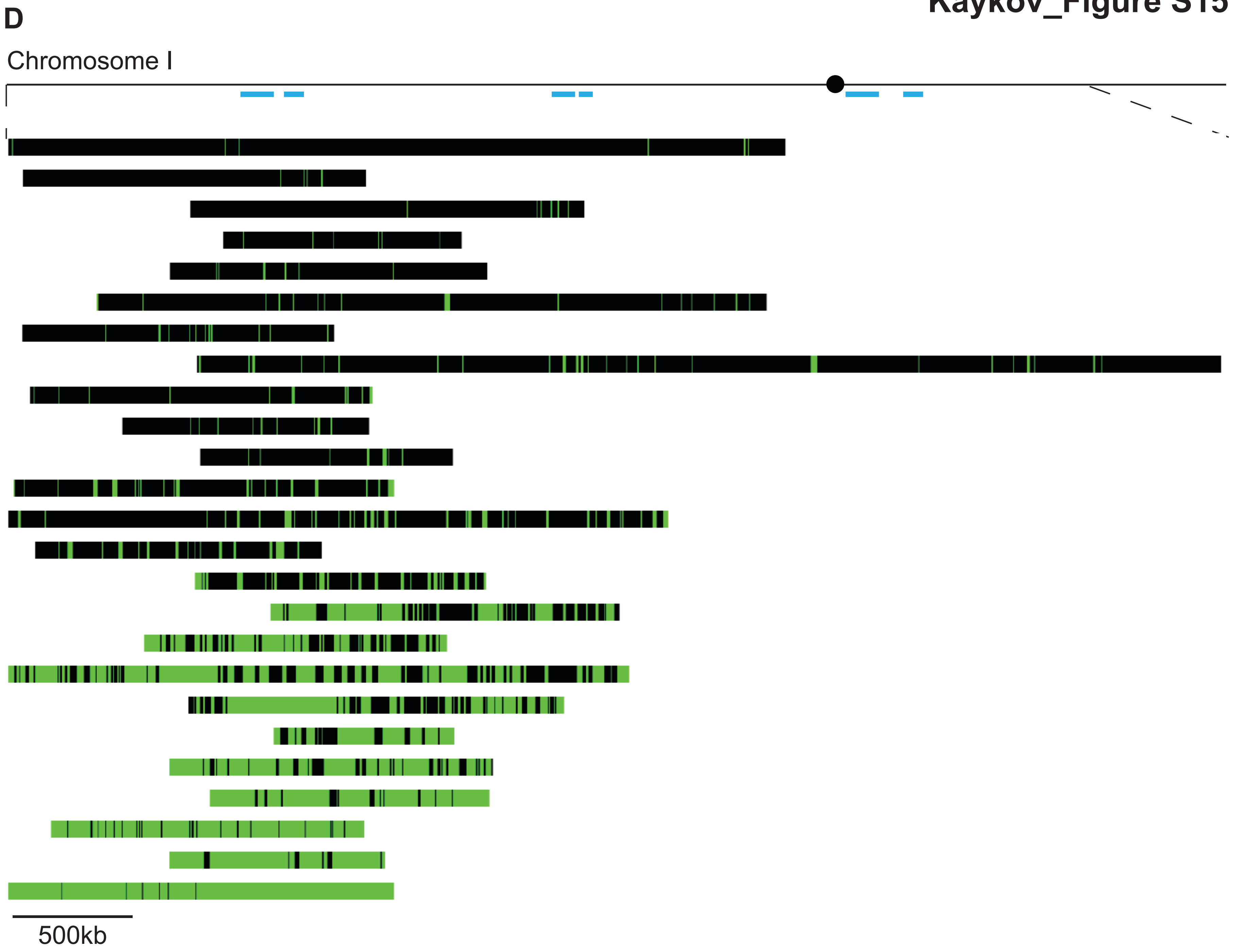
D
Chromosome I
500kb

Kaykov_Figure S15

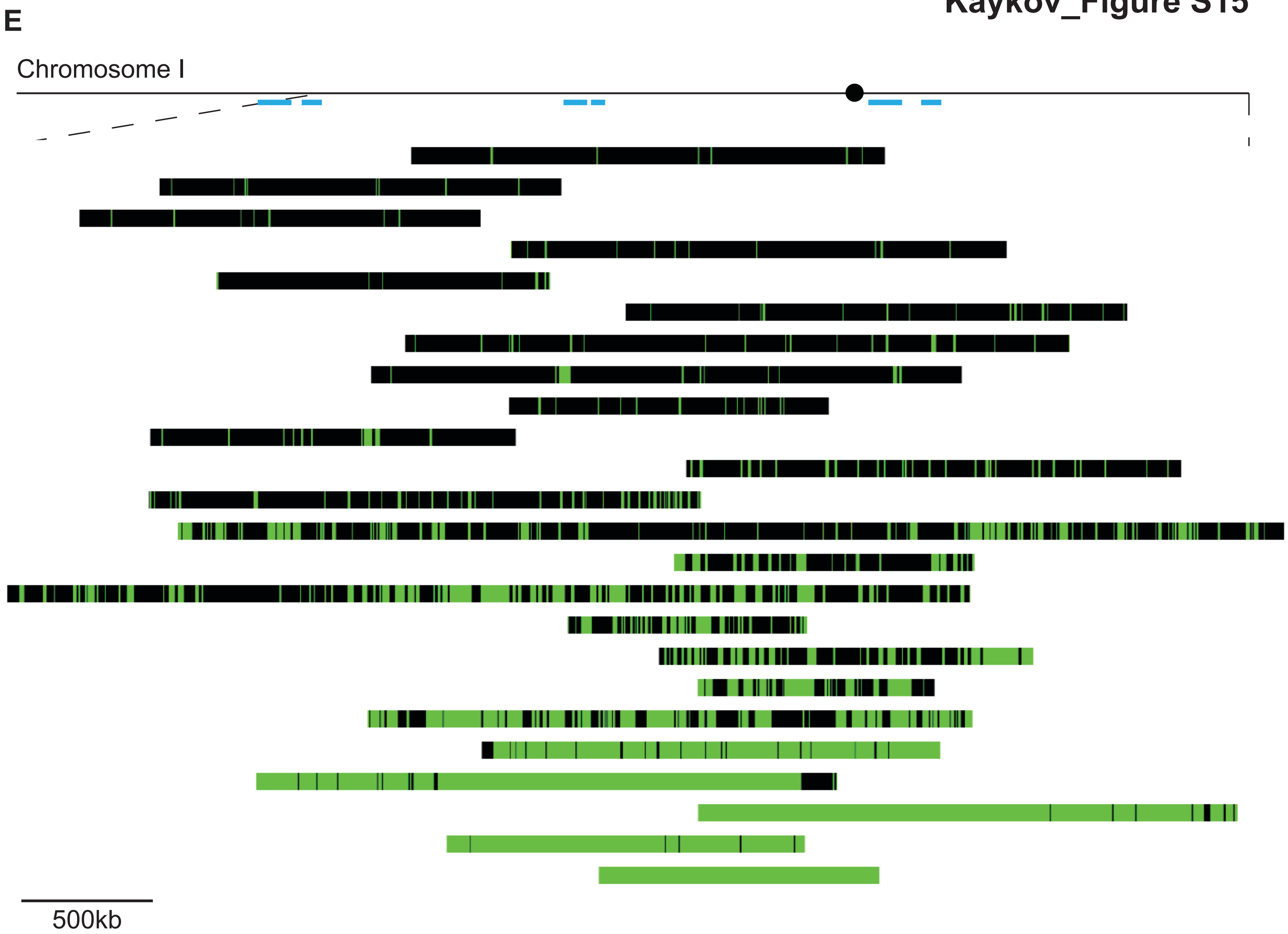

Kaykov_Figure S15

F

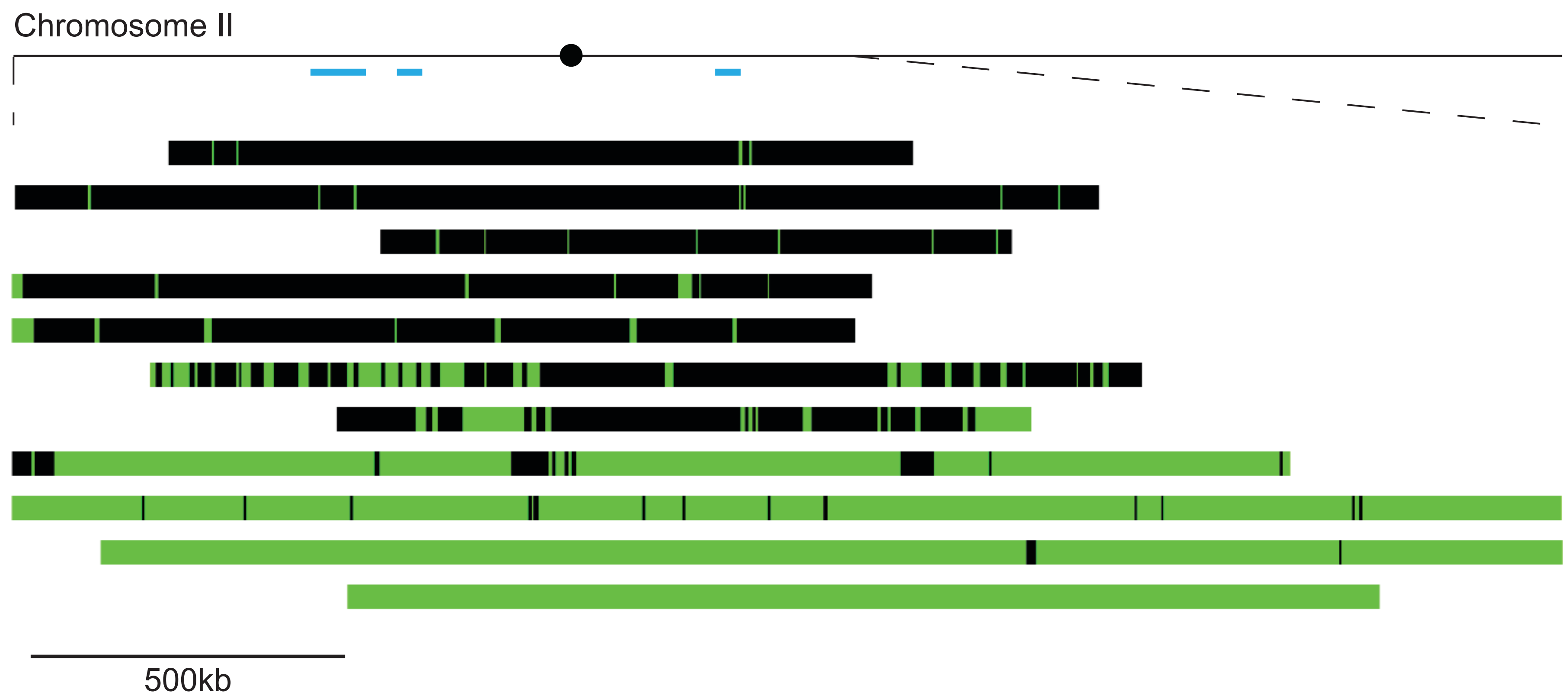

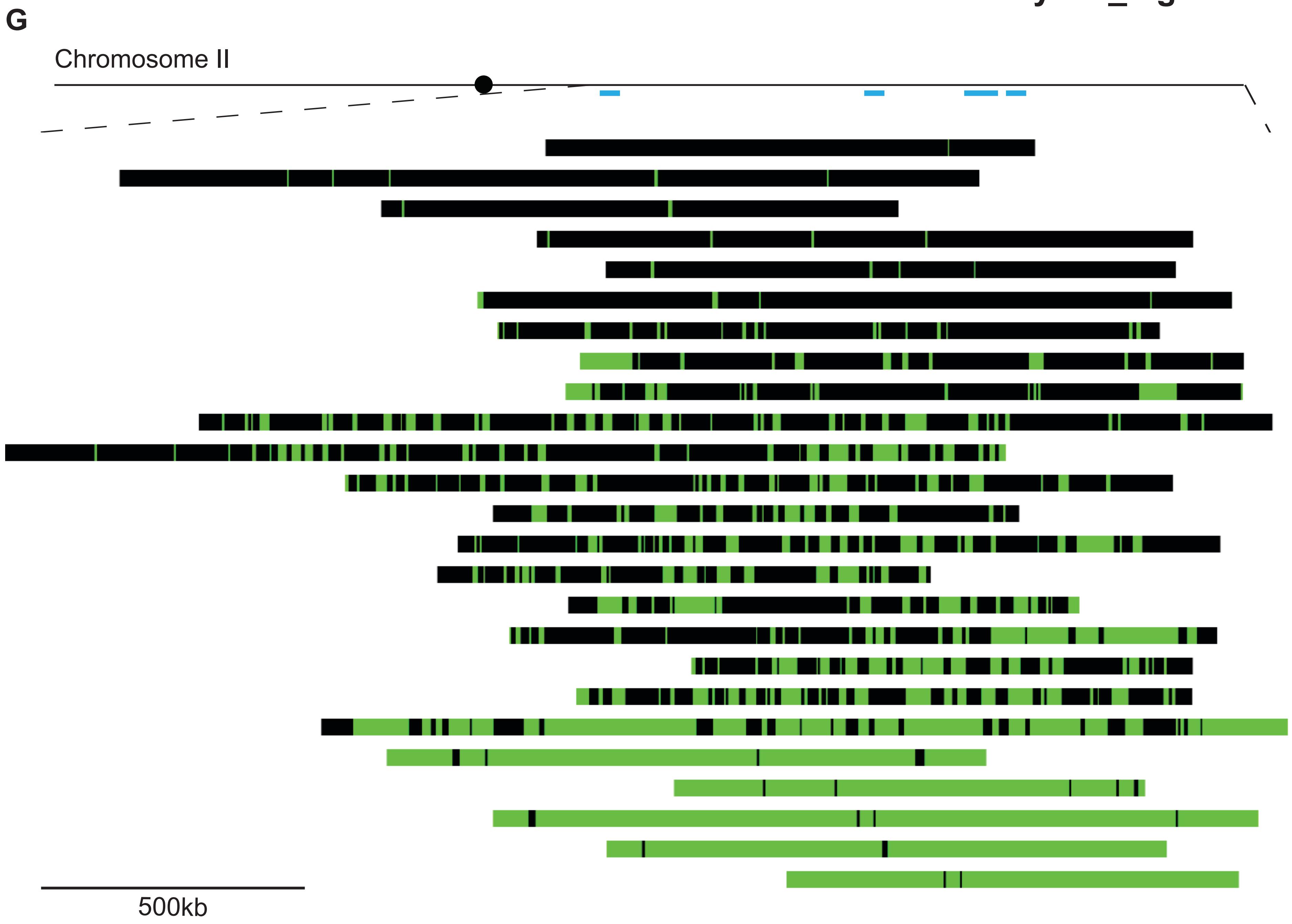
G
Chromosome II
500kb

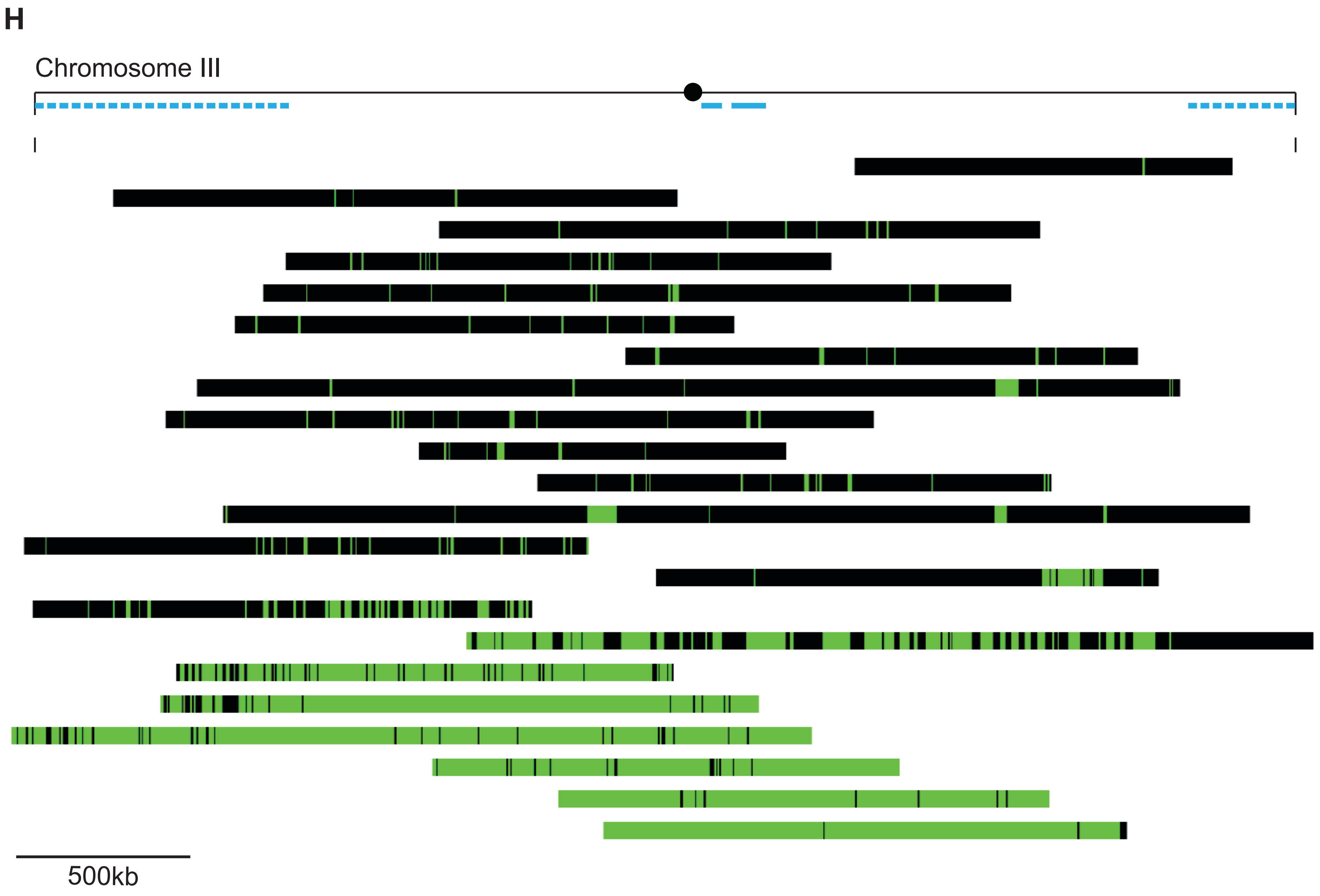
H
Chromosome III
500kb

I

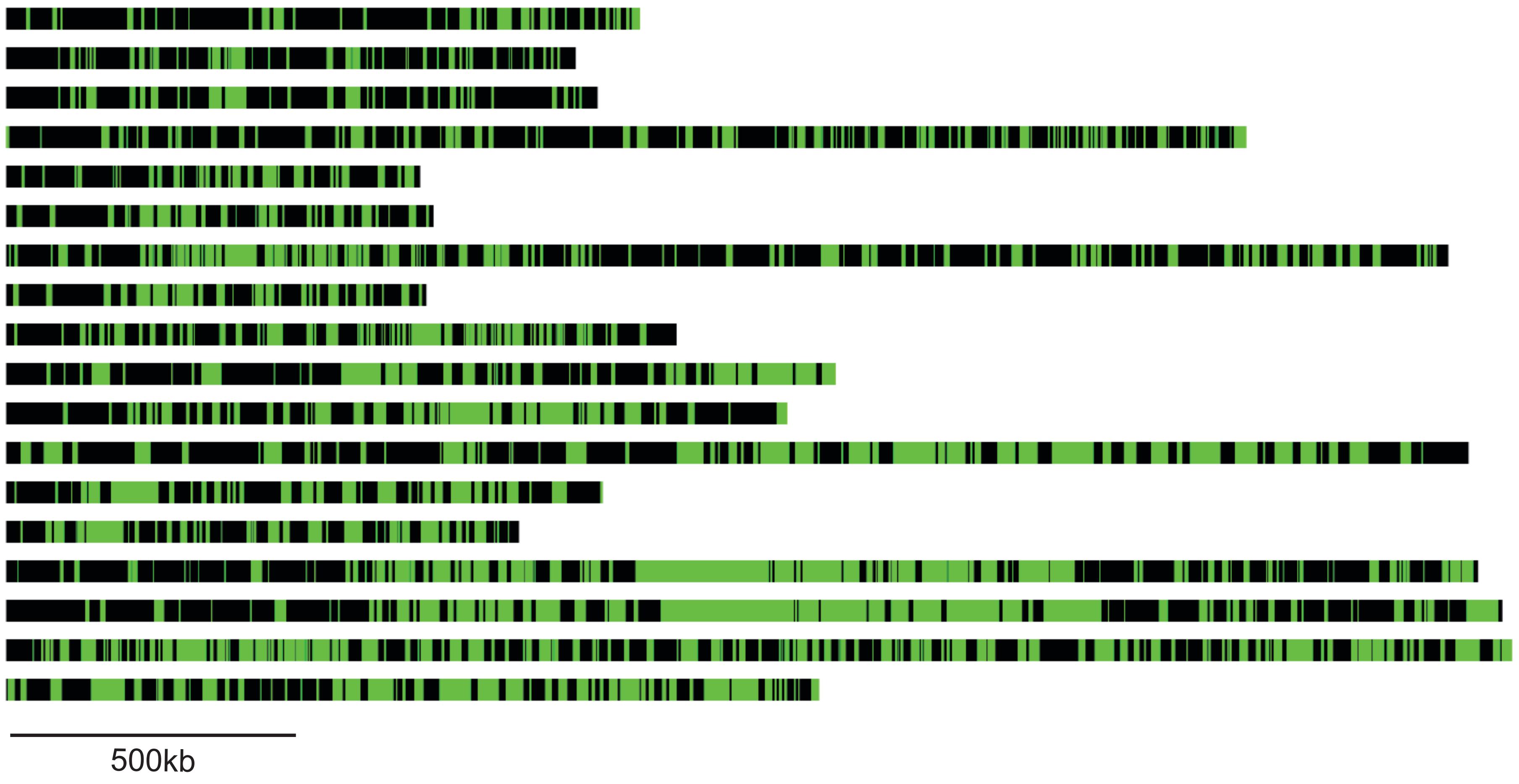

Kaykov_Figure S15

Figure S15. Single DNA molecules analysed in this study

(A) and (B) Single DNA molecules extracted from synchronous cell population advanced to 20% and 50% DNA replication respectively (estimated for the population of cells from FACS profile). (C) Single DNA molecules extracted from synchronous cell population pulsed with BrdU (green) in G1 and chased with EdU (red) in mid S-phase. DNA molecules are not aligned to chromosomal sequences since FISH probes were not hybridised. (D) and (E) Single DNA molecules aligned to the corresponding sequence on chromosome I (5.6Mb), (F) and (G) chromosome II (4.5Mb) and (H) chromosome III (~3.5Mb). Chromosomes, centromeres and the position of FISH probes (blue lines) are represented schematically. The position of each single DNA molecule is estimated relative to the position of hybridised FISH probes on each of the molecules. The blue dashed lines at both ends of chromosomes III correspond to FISH probe hybridised to rDNA repeats. (I) Single DNA molecules extracted from *cdc25-22clr3∆clr4∆* synchronous cell population advanced to 50% DNA replication (estimated for the population of cells from FACS profile). In all panels molecules are ordered by increasing percentage of DNA replication. The length (kb) of un-replicated DNA segments and replication tracks in addition to the left end position for all DNA molecules represented in panels A-I are listed in supplementary Table S2.

Supplemental References

Meister, P., Poidevin, M., Francesconi, S., Tratner, I., Zarzov, P., and Baldacci, G. (2003). Nuclear factories for signalling and repairing DNA double strand breaks in living fission yeast. Nucleic acids research *31*, 5064-5073.

Sivakumar, S., Porter-Goff, M., Patel, P.K., Benoit, K., and Rhind, N. (2004). In vivo labeling of fission yeast DNA with thymidine and thymidine analogs. Methods *33*, 213-219.
