# Supplementary material for: The spatial and temporal organization of origin firing during the S-phase of fission yeast
Source: Genome Res. 2015 Mar;25(3):391–401. doi: 10.1101/gr.180372.114 (PMC4352884; doi:10.1101/gr.180372.114)
Supplement: Supplemental Material [file supp_gr.180372.114_Table_S1.ps]

TABLE S1. List of strains used in this study

Strain	Genotype	Source
PN10597	*h- leu1-32::pFS181[Padh1-hENT1 leu1+] his7-336 pJL218[Padh1-hsvTK his7+]*	derived from YFS240 Sivakumar et al., 2004
PN10598	*h-(Msmt-0) leu1-32::pFS181[Padh1-hENT1 leu1+] his7-336 pJL218[Padh1-hsvTK his7+] clr3Δ::KanR*	This study
PN10599	*h-(Msmt-0) leu1-32::pFS181[Padh1-hENT1 leu1+] his7-336 pJL218[Padh1-hsvTK his7+] clr4Δ::LEU2*	This study
PN10600	*h-(Msmt-0) leu1-32::pFS181[Padh1-hENT1 leu1+] his7-336 pJL218[Padh1-hsvTK his7+] clr3Δ::KanR clr4Δ::LEU2*	This study
PN10602	*h-(Msmt-0) leu1-32::pFS181[Padh1-hENT1 leu1+] his7-336 pJL218[Padh1-hsvTK his7+] hos2Δ::LEU2*	This study
PN10603	*h-(Msmt-0) leu1-32::pFS181[Padh1-hENT1 leu1+] his7-336 pJL218[Padh1-hsvTK his7+] sir2Δ::KanR*	This study
PN10604	*h- leu1-32::pFS181[Padh1-hENT1 leu1+] his7-336 pJL218[Padh1-hsvTK his7+] ura4-D18 swi6Δ::ura4+*	This study
PN10605	*h- leu1-32::pFS181[Padh1-hENT1 leu1+] his7-336 pJL218[Padh1-hsvTK his7+] ura4-D18 cds1Δ::ura4+*	This study
PN10606	*h-(Msmt-0) leu1-32::pFS181[Padh1-hENT1 leu1+] his7-336 pJL218[Padh1-hsvTK his7+] rad21-45*	This study
PN10499	*h-(Msmt-0) leu1-32::pFS181[Padh1-hENT1 leu1+] his7-336 pJL218[Padh1-hsvTK his7+] cdc25-22*	This study
PN10607	*h-(Msmt-0) leu1-32::pFS181[Padh1-hENT1 leu1+] his7-336 pJL218[Padh1-hsvTK his7+] clr3Δ::KanR clr4Δ::LEU2 cdc25-22*	This study
SP150	*h+ leu1-32 ura4::Ppcna1-GFP-pcna1*	Meister et al., 2003
PN10608	*h+ leu1-32 ura4::Ppcna1-GFP-pcna1 clr3Δ::KanR*	This study
PN10609	*h+ leu1-32 ura4::Ppcna1-GFP-pcna1 clr4Δ::LEU2*	This study
PN10610	*h+ leu1-32 ura4::Ppcna1-GFP-pcna1 clr3Δ::KanR clr4Δ::LEU2*	This study
